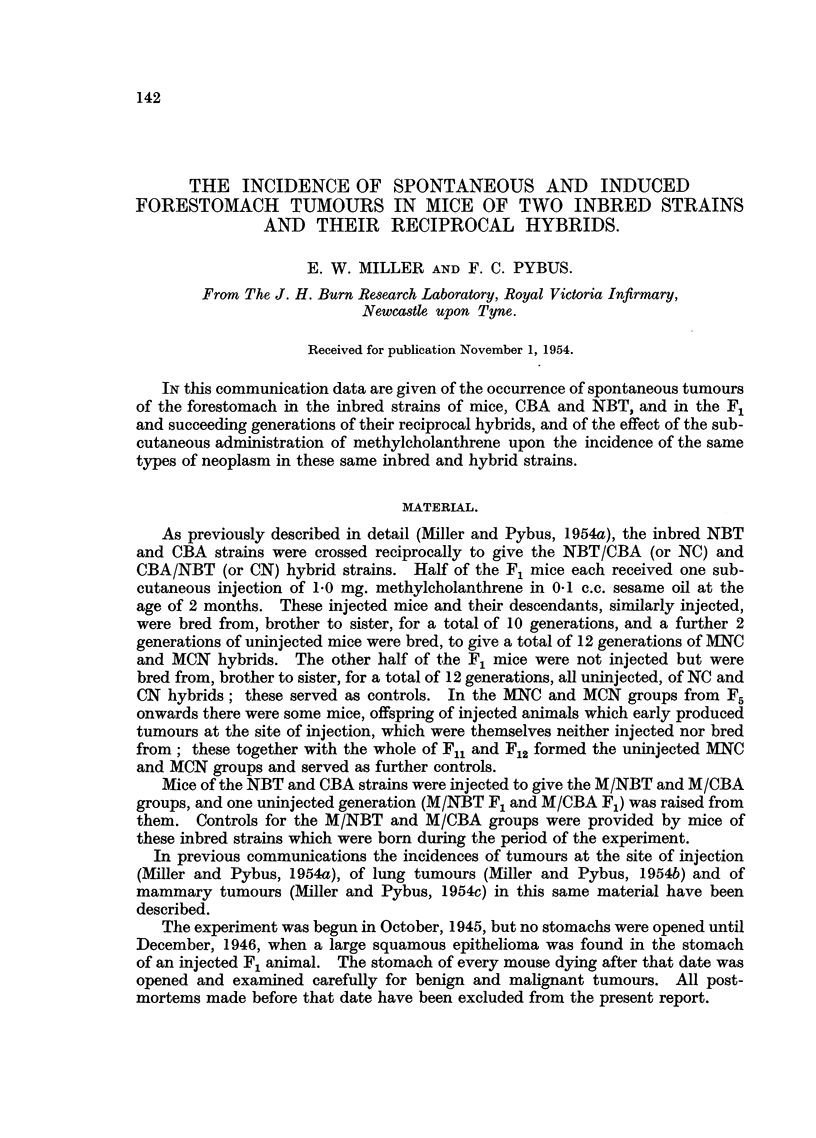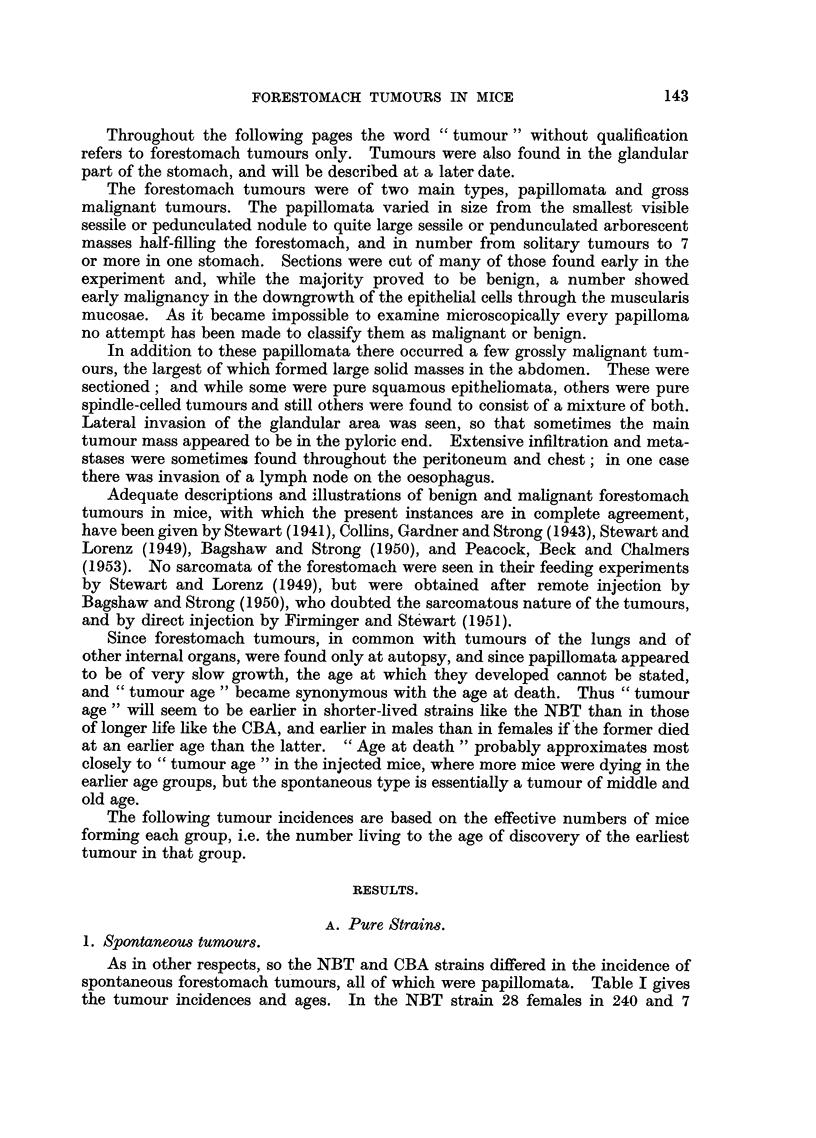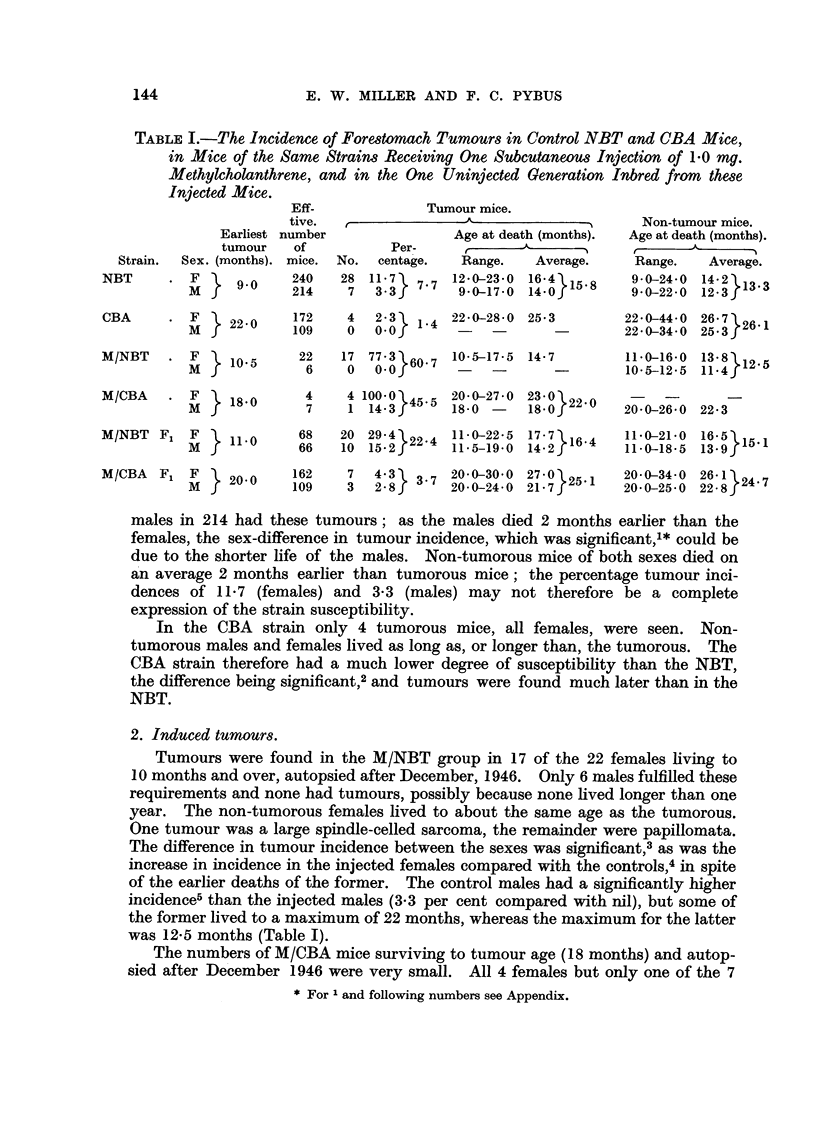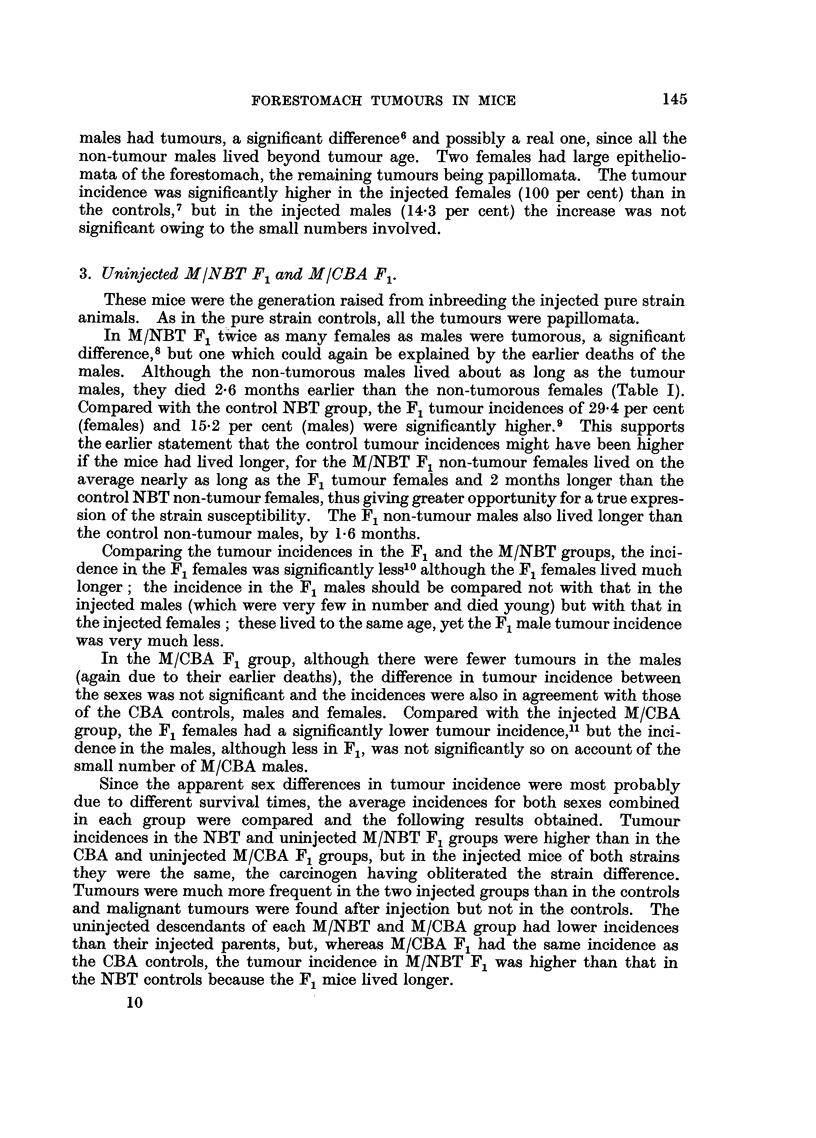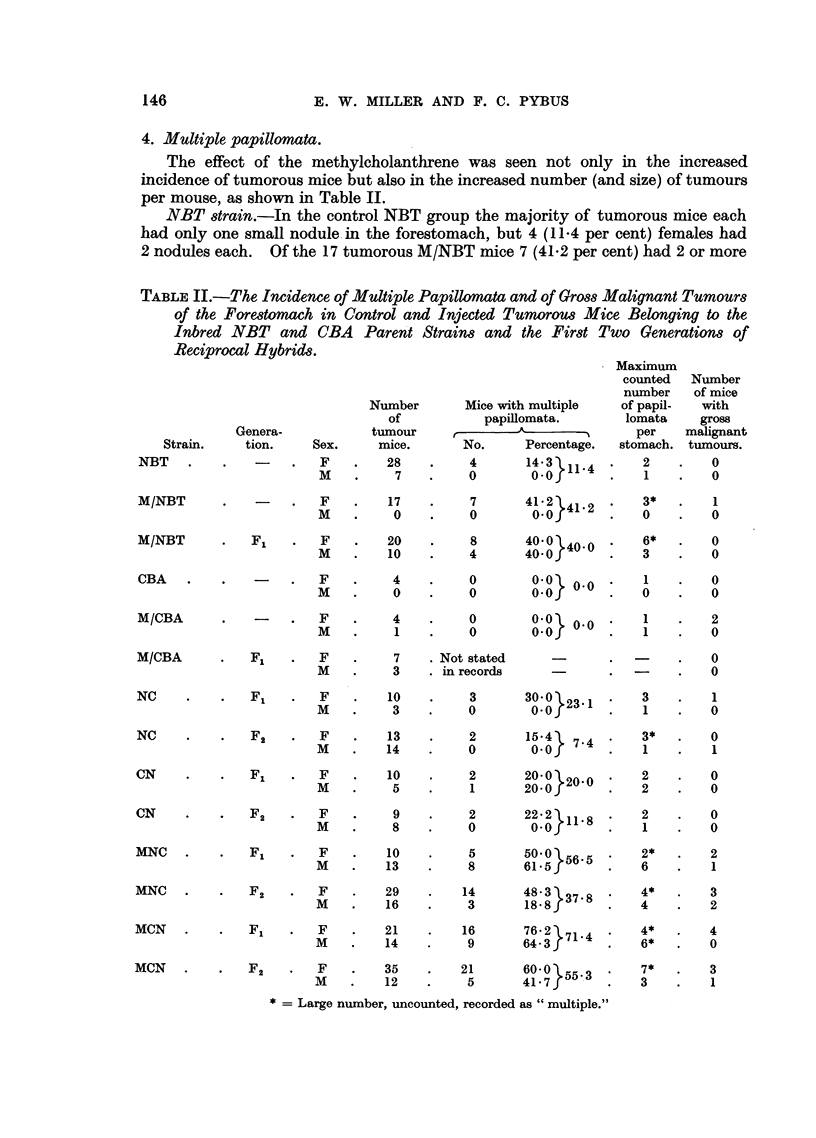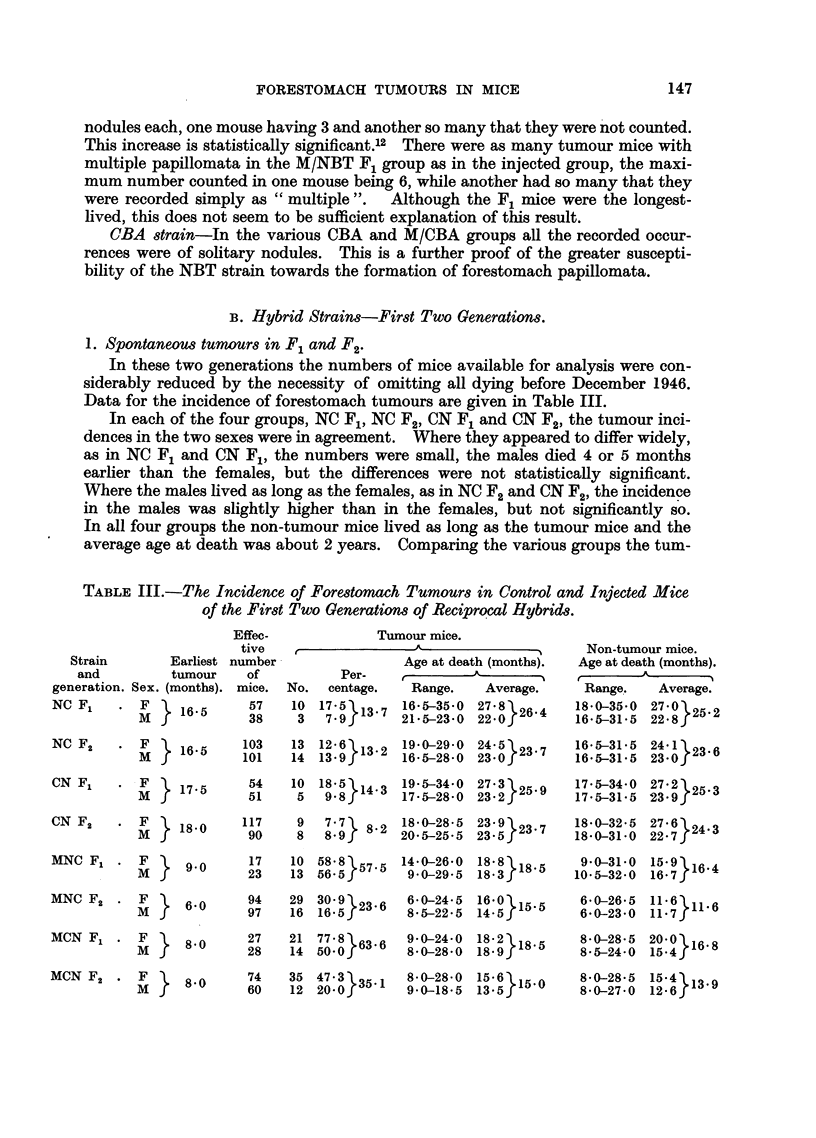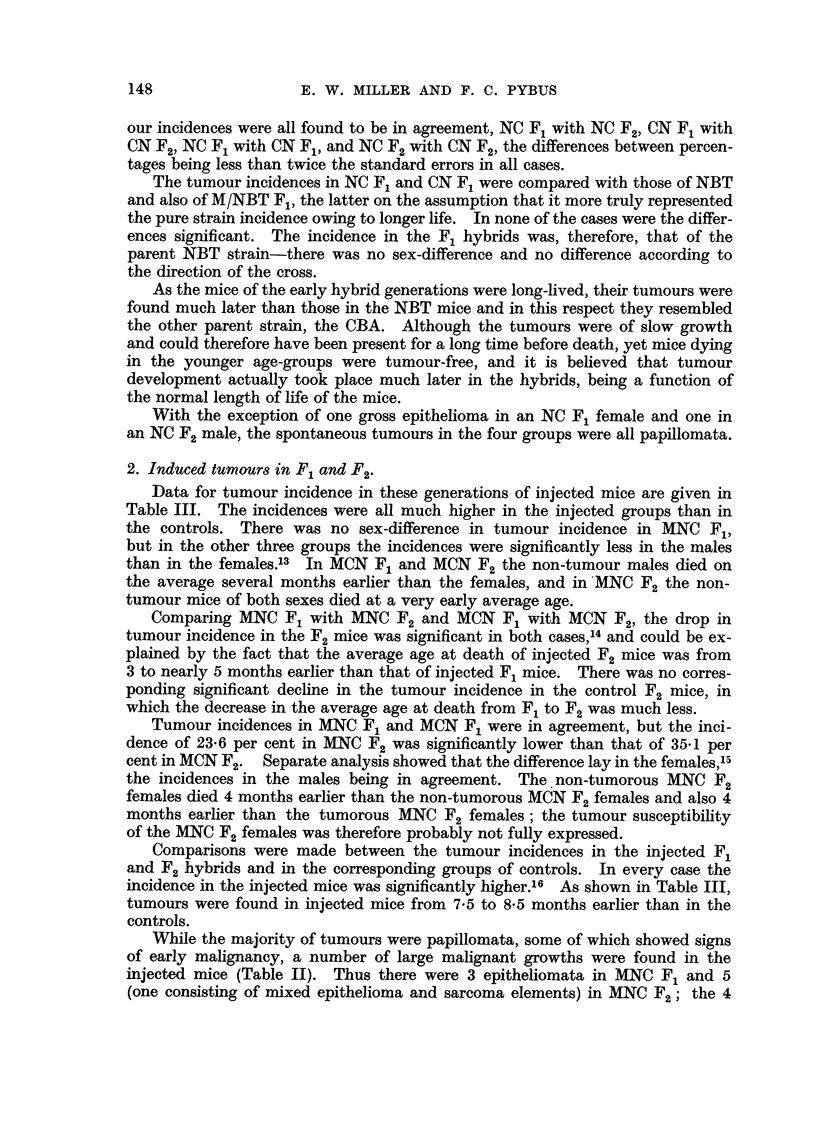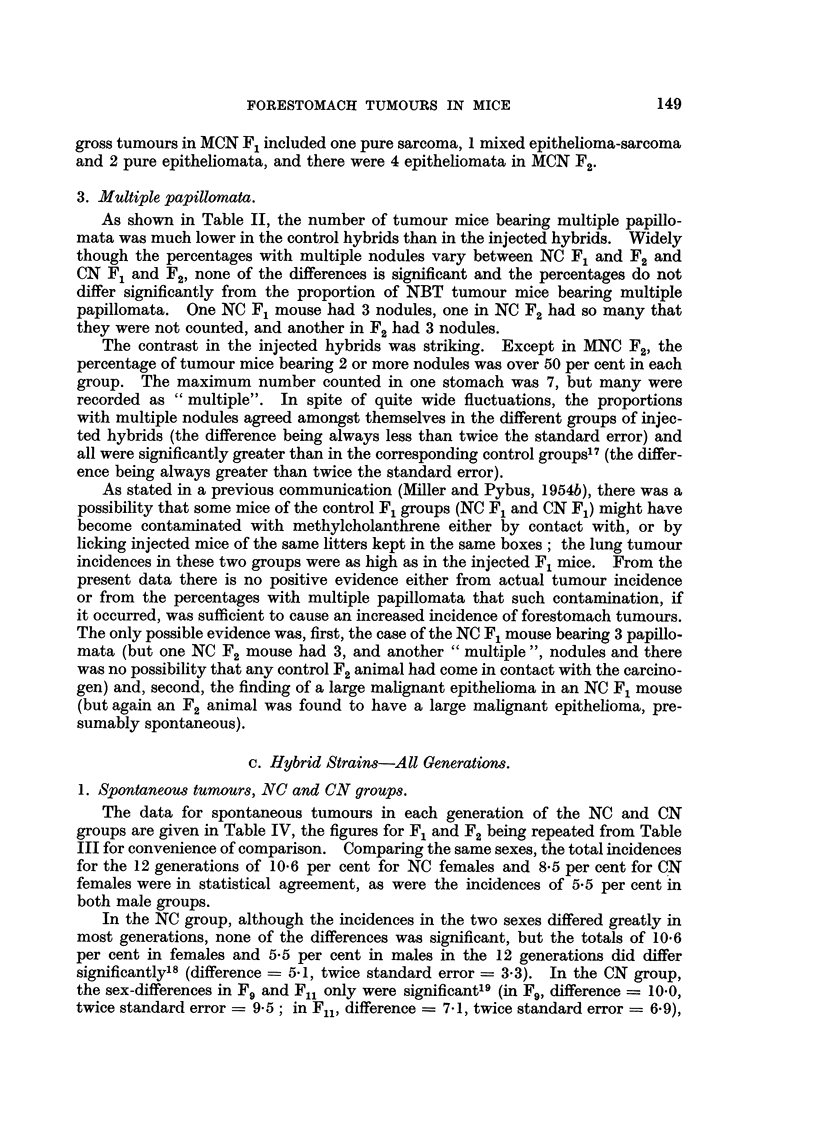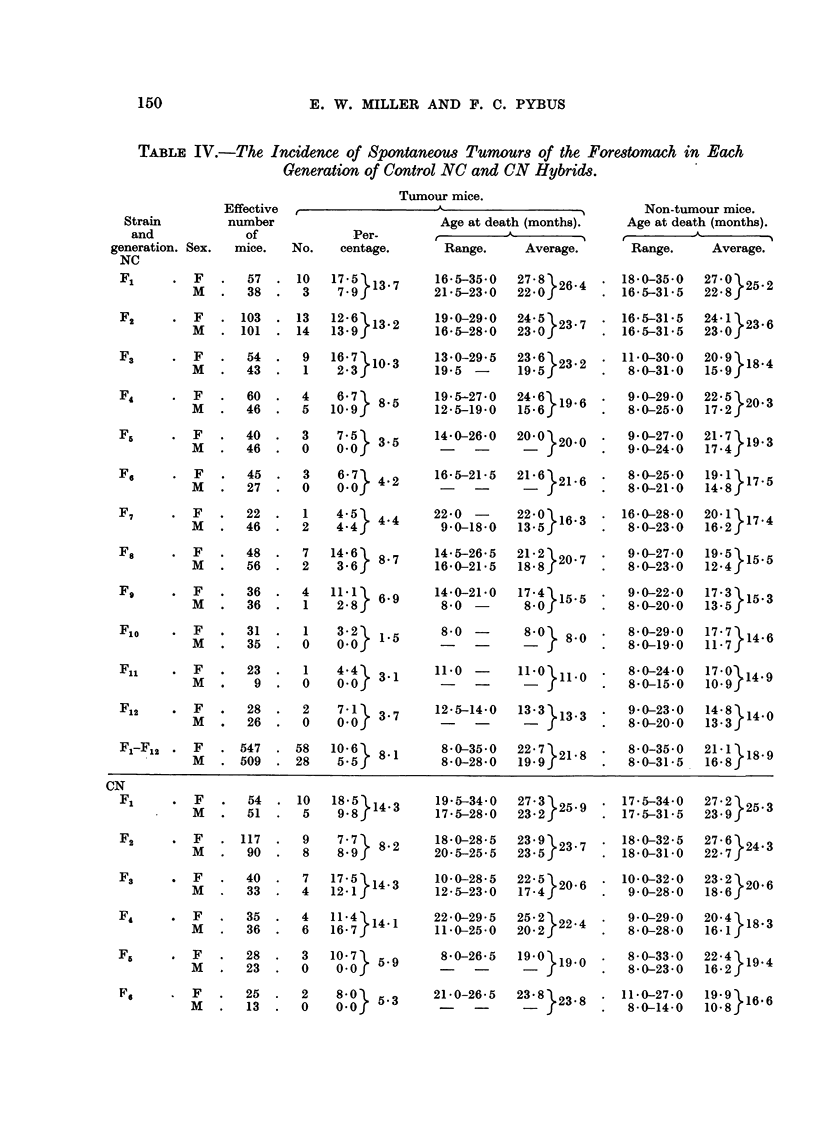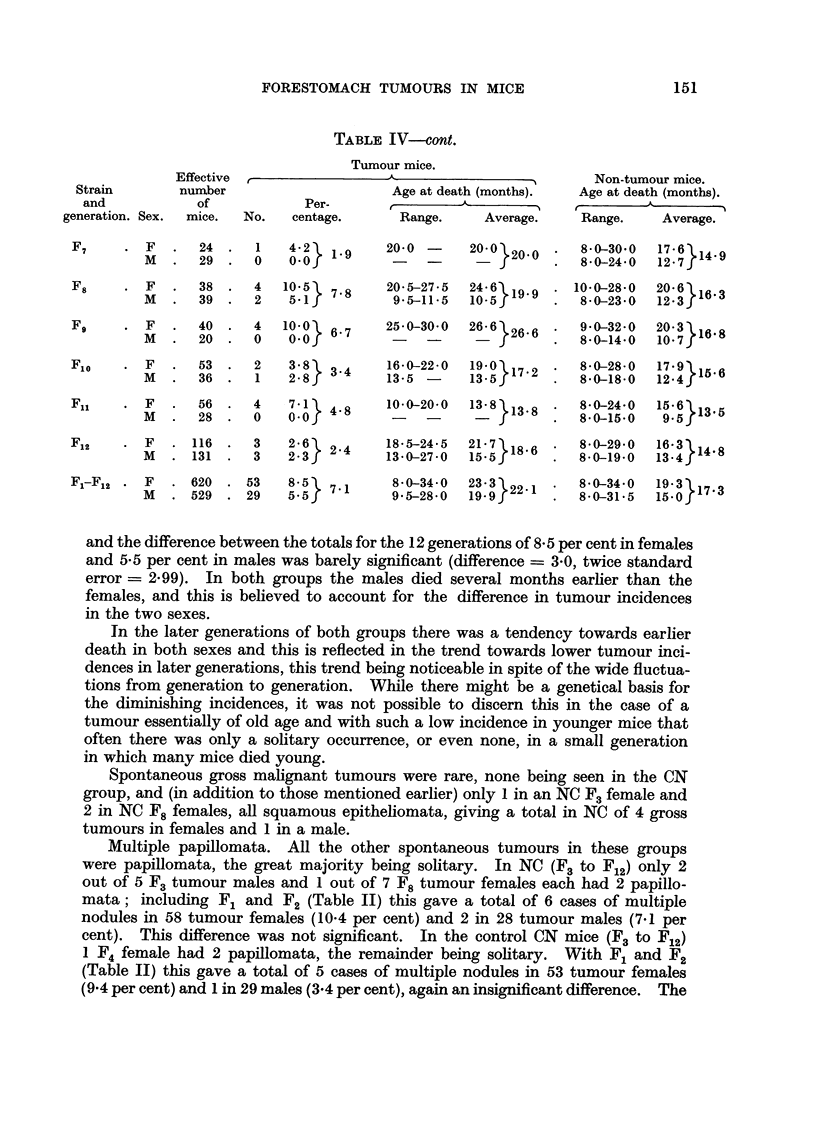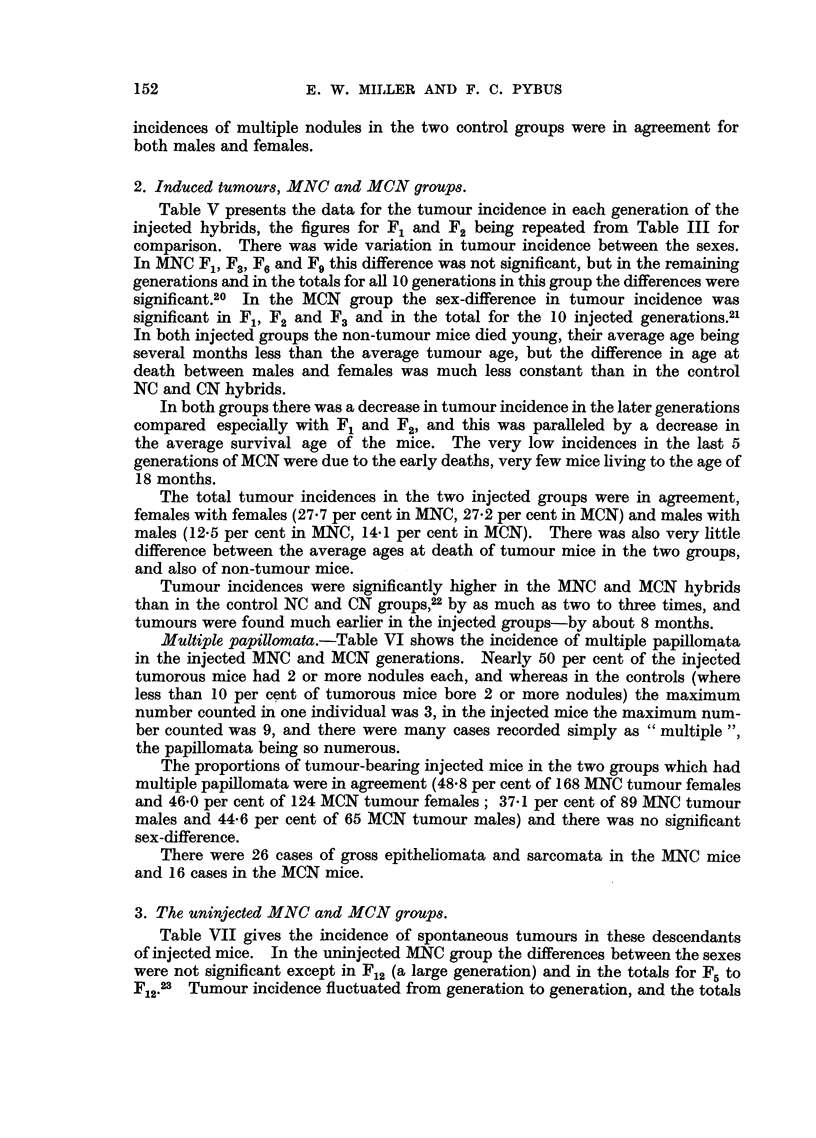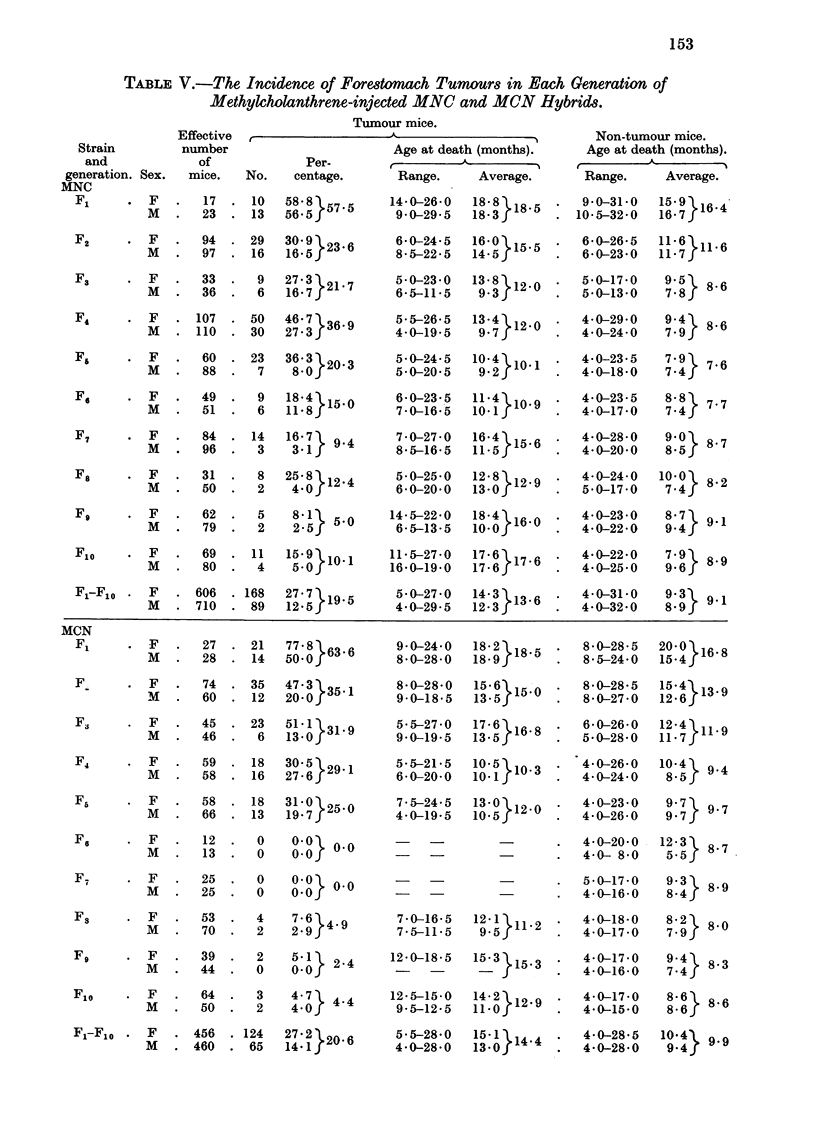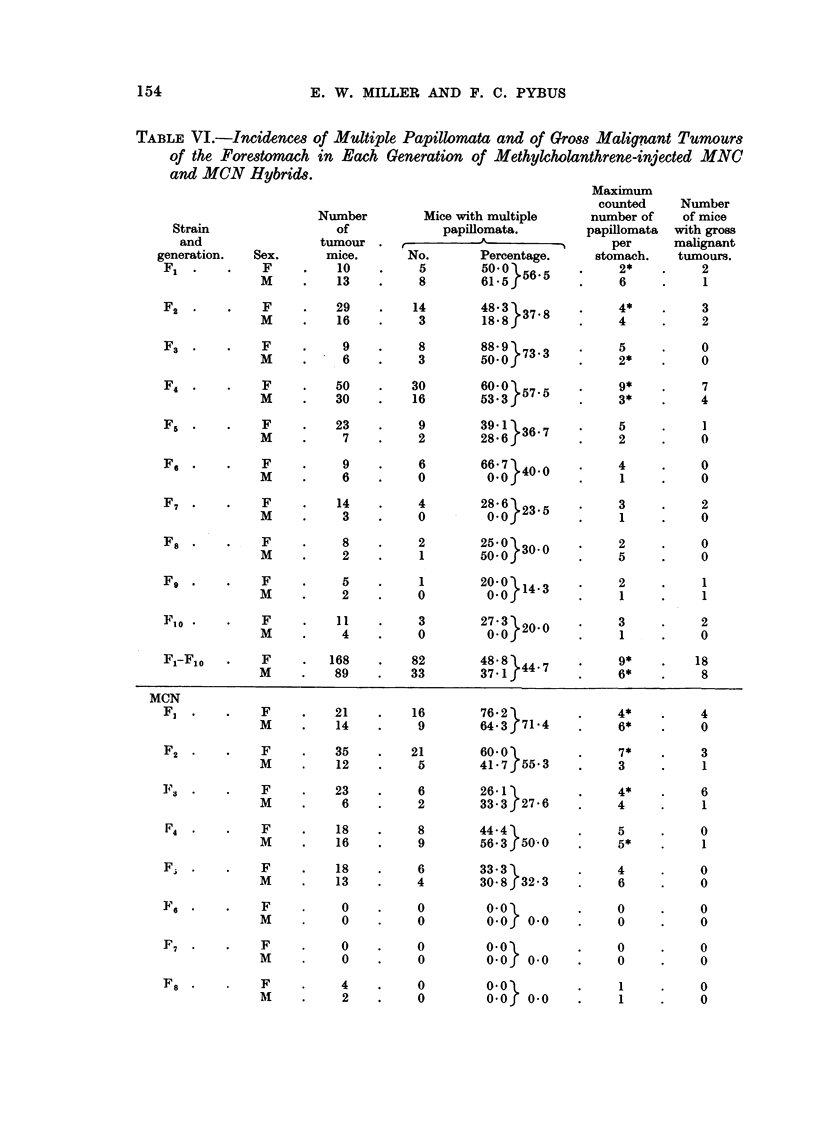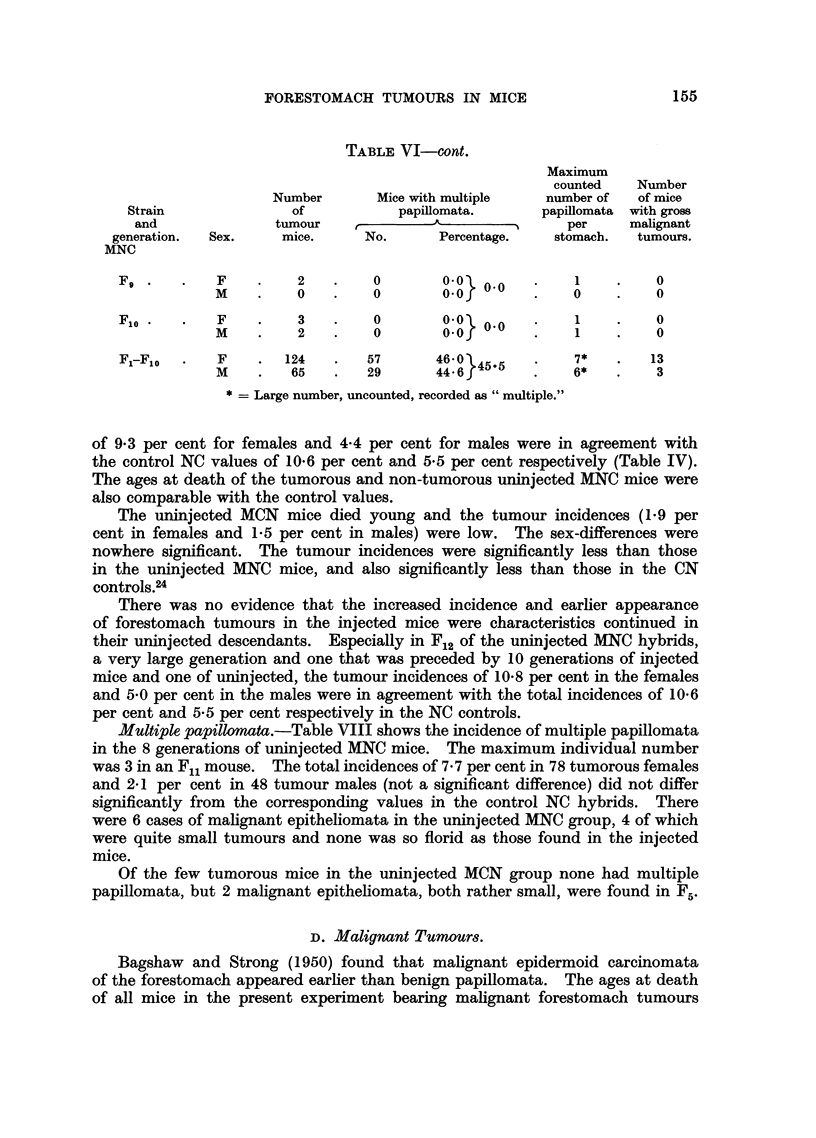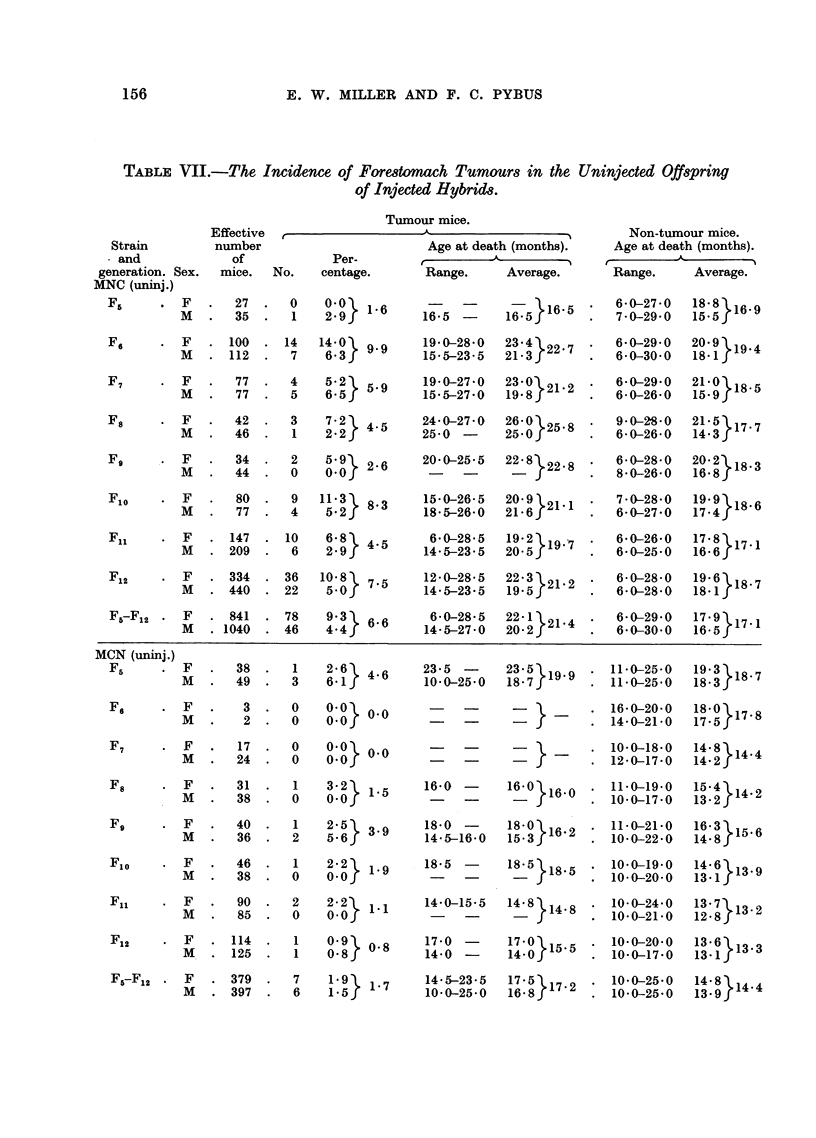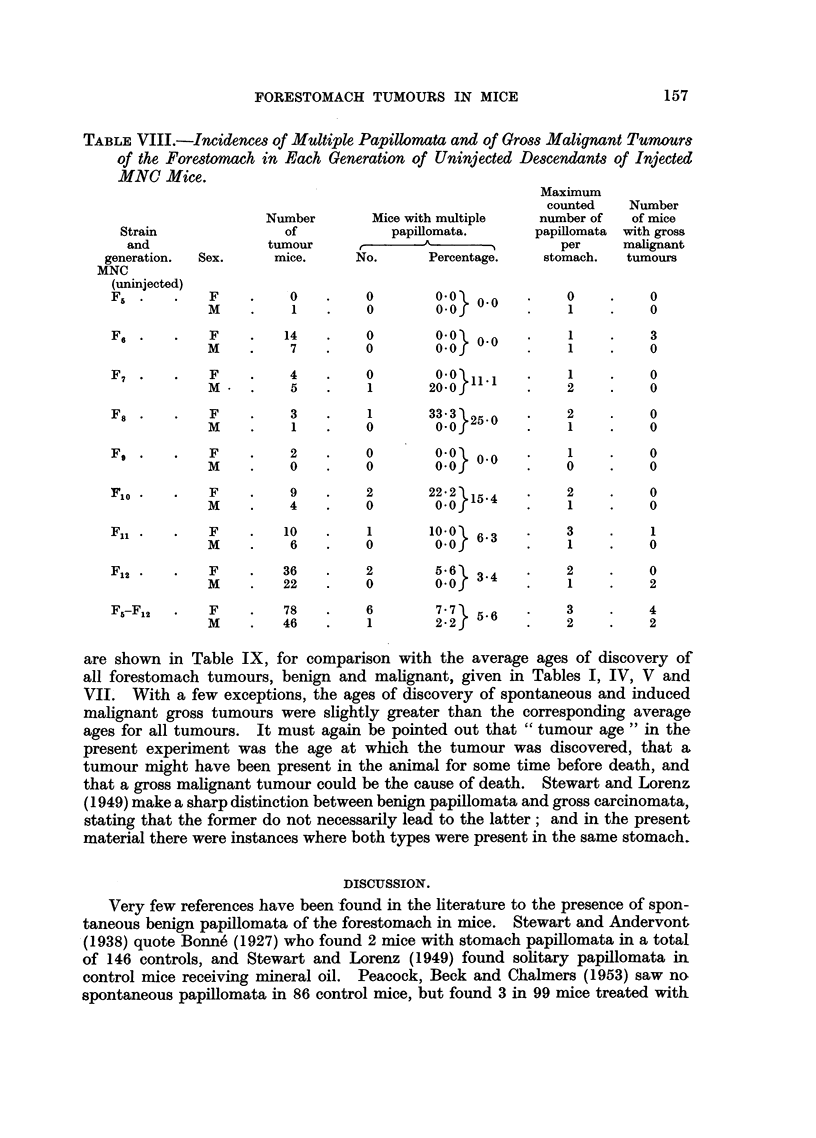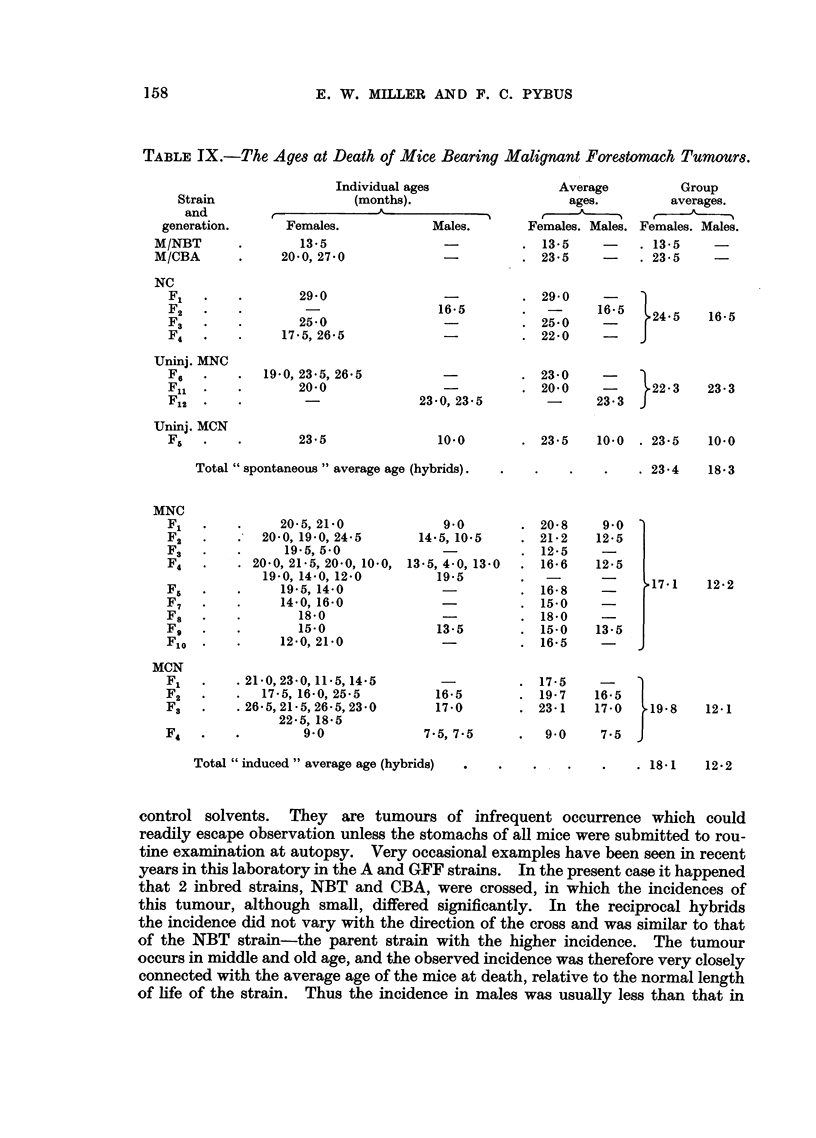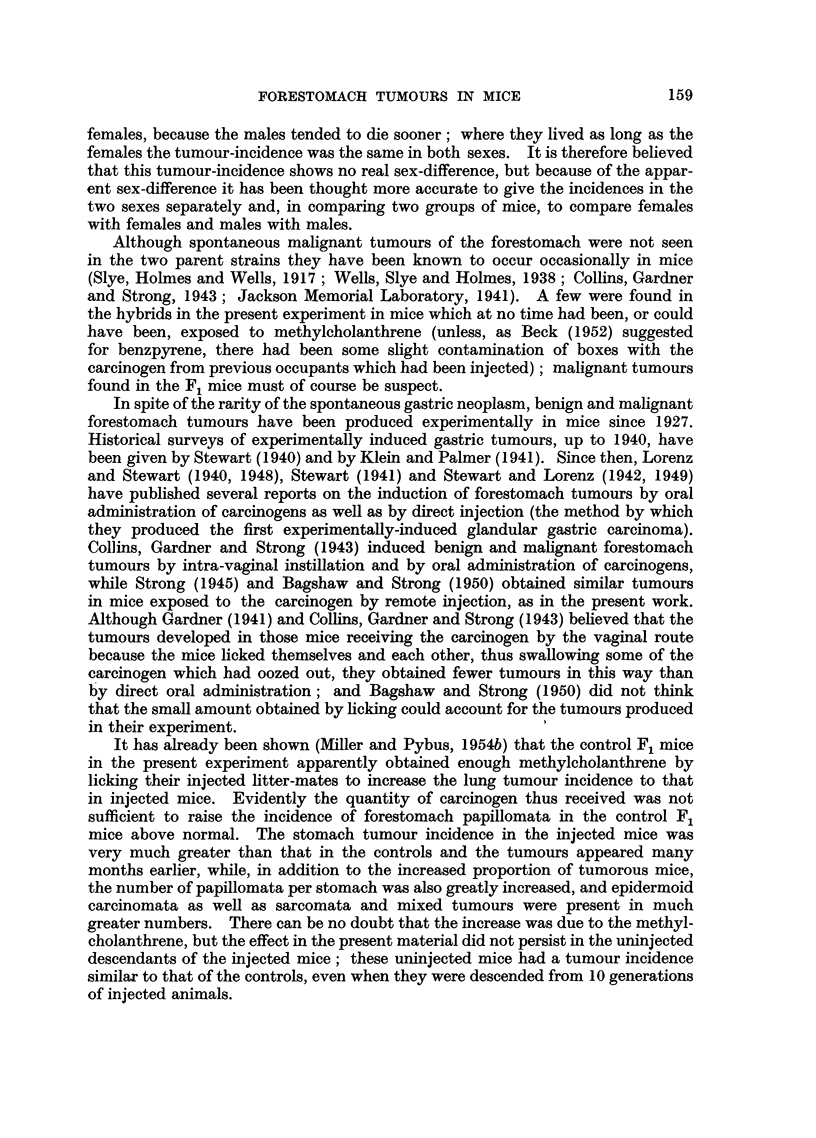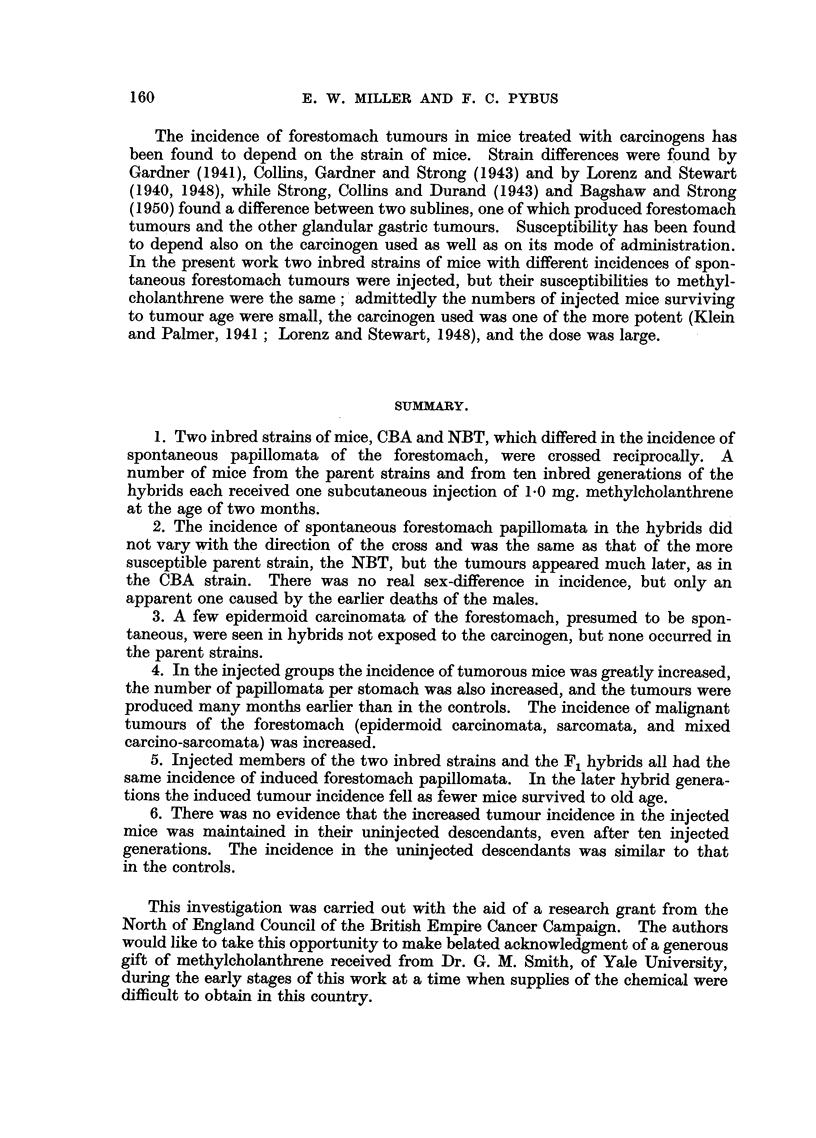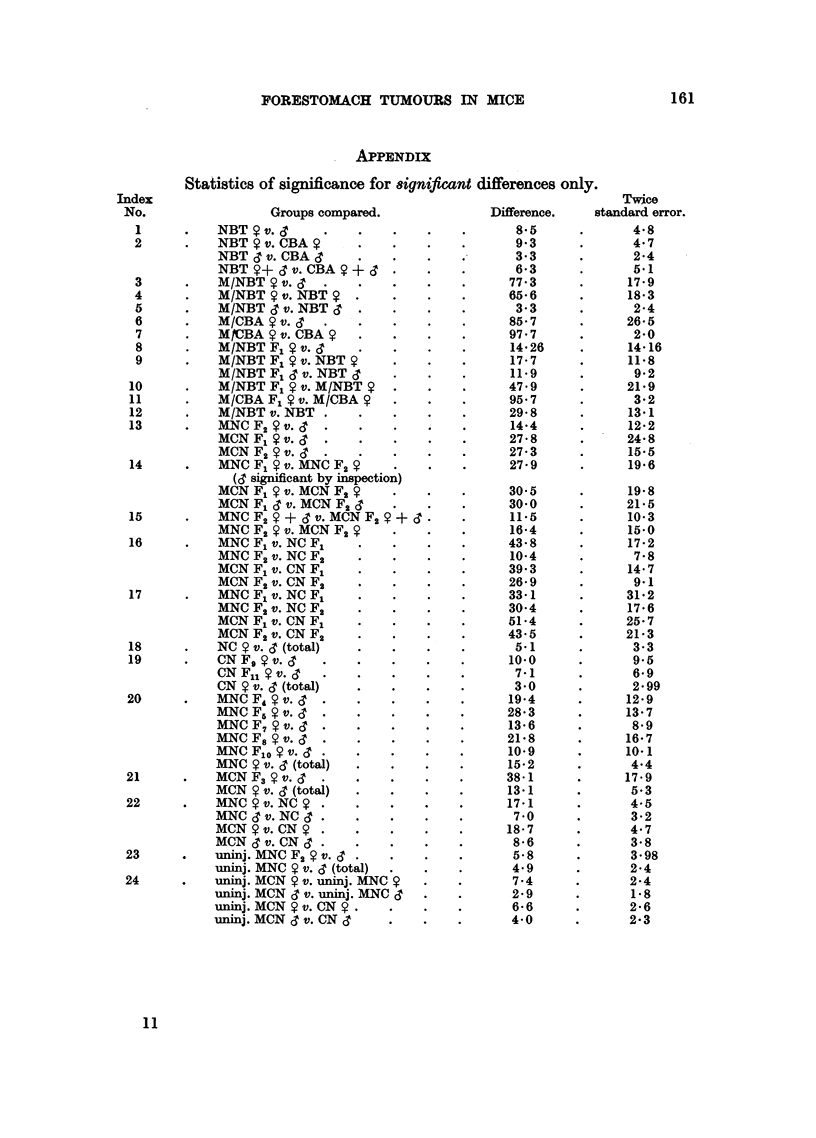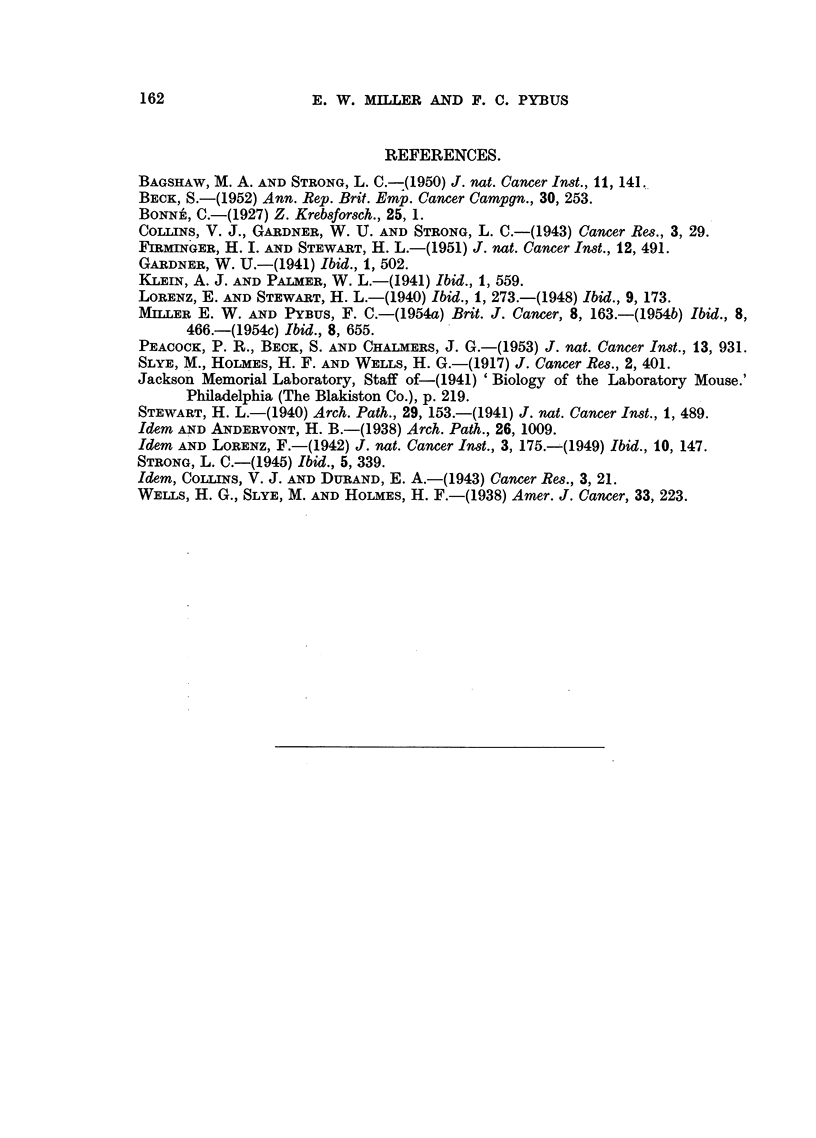# The Incidence of Spontaneous and Induced Forestomach Tumours in Mice of Two Inbred Strains and their Reciprocal Hybrids

**DOI:** 10.1038/bjc.1955.11

**Published:** 1955-03

**Authors:** E. W. Miller, F. C. Pybus


### THE INCIDENCE OF SPONTANEOUS AND INDUCED FORESTOMACH TUMOURS IN MICE OF TWO INBRED STRAINS AND THEIR RECIPROCAL HYBRIDS.

E. W. MILLER AND F. C. PYBUS.

*From The J. H. Burn Research Laboratory, Royal Victoria Infirmary, Newcastle upon Tyne.*

Received for publication November 1, 1954.

IN this communication data are given of the occurrence of spontaneous tumours of the forestomach in the inbred strains of mice, CBA and NBT, and in the $F_1$ and succeeding generations of their reciprocal hybrids, and of the effect of the subcutaneous administration of methylcholanthrene upon the incidence of the same types of neoplasm in these same inbred and hybrid strains.

### MATERIAL.

As previously described in detail (Miller and Pybus, 1954*a*), the inbred NBT and CBA strains were crossed reciprocally to give the NBT/CBA (or NC) and CBA/NBT (or CN) hybrid strains. Half of the $F_1$ mice each received one subcutaneous injection of 1·0 mg. methylcholanthrene in 0·1 c.c. sesame oil at the age of 2 months. These injected mice and their descendants, similarly injected, were bred from, brother to sister, for a total of 10 generations, and a further 2 generations of uninjected mice were bred, to give a total of 12 generations of MNC and MCN hybrids. The other half of the $F_1$ mice were not injected but were bred from, brother to sister, for a total of 12 generations, all uninjected, of NC and CN hybrids; these served as controls. In the MNC and MCN groups from $F_5$ onwards there were some mice, offspring of injected animals which early produced tumours at the site of injection, which were themselves neither injected nor bred from; these together with the whole of $F_{11}$ and $F_{12}$ formed the uninjected MNC and MCN groups and served as further controls.

Mice of the NBT and CBA strains were injected to give the M/NBT and M/CBA groups, and one uninjected generation (M/NBT $F_1$ and M/CBA $F_1$) was raised from them. Controls for the M/NBT and M/CBA groups were provided by mice of these inbred strains which were born during the period of the experiment.

In previous communications the incidences of tumours at the site of injection (Miller and Pybus, 1954*a*), of lung tumours (Miller and Pybus, 1954*b*) and of mammary tumours (Miller and Pybus, 1954*c*) in this same material have been described.

The experiment was begun in October, 1945, but no stomachs were opened until December, 1946, when a large squamous epithelioma was found in the stomach of an injected $F_1$ animal. The stomach of every mouse dying after that date was opened and examined carefully for benign and malignant tumours. All postmortems made before that date have been excluded from the present report.

Throughout the following pages the word " tumour " without qualification refers to forestomach tumours only. Tumours were also found in the glandular part of the stomach, and will be described at a later date.

The forestomach tumours were of two main types, papillomata and gross malignant tumours. The papillomata varied in size from the smallest visible sessile or pedunculated nodule to quite large sessile or pendunculated arborescent masses half-filling the forestomach, and in number from solitary tumours to 7 or more in one stomach. Sections were cut of many of those found early in the experiment and, while the majority proved to be benign, a number showed early malignancy in the downgrowth of the epithelial cells through the muscularis mucosae. As it became impossible to examine microscopically every papilloma no attempt has been made to classify them as malignant or benign.

In addition to these papillomata there occurred a few grossly malignant tumours, the largest of which formed large solid masses in the abdomen. These were sectioned ; and while some were pure squamous epitheliomata, others were pure spindle-celled tumours and still others were found to consist of a mixture of both. Lateral invasion of the glandular area was seen, so that sometimes the main tumour mass appeared to be in the pyloric end. Extensive infiltration and metastases were sometimes found throughout the peritoneum and chest ; in one case there was invasion of a lymph node on the oesophagus.

Adequate descriptions and illustrations of benign and malignant forestomach tumours in mice, with which the present instances are in complete agreement, have been given by Stewart (1941), Collins, Gardner and Strong (1943), Stewart and Lorenz (1949), Bagshaw and Strong (1950), and Peacock, Beck and Chalmers (1953). No sarcomata of the forestomach were seen in their feeding experiments by Stewart and Lorenz (1949), but were obtained after remote injection by Bagshaw and Strong (1950), who doubted the sarcomatous nature of the tumours, and by direct injection by Firminger and Stewart (1951).

Since forestomach tumours, in common with tumours of the lungs and of other internal organs, were found only at autopsy, and since papillomata appeared to be of very slow growth, the age at which they developed cannot be stated, and " tumour age " became synonymous with the age at death. Thus " tumour age " will seem to be earlier in shorter-lived strains like the NBT than in those of longer life like the CBA, and earlier in males than in females if the former died at an earlier age than the latter. " Age at death " probably approximates most closely to " tumour age " in the injected mice, where more mice were dying in the earlier age groups, but the spontaneous type is essentially a tumour of middle and old age.

The following tumour incidences are based on the effective numbers of mice forming each group, i.e. the number living to the age of discovery of the earliest tumour in that group.

### RESULTS.

### A. *Pure Strains.*

1. *Spontaneous tumours.*

As in other respects, so the NBT and CBA strains differed in the incidence of spontaneous forestomach tumours, all of which were papillomata. Table I gives the tumour incidences and ages. In the NBT strain 28 females in 240 and 7

TABLE I.—*The Incidence of Forestomach Tumours in Control NBT and CBA Mice, in Mice of the Same Strains Receiving One Subcutaneous Injection of* 1·0 *mg. Methylcholanthrene, and in the One Uninjected Generation Inbred from these Injected Mice.*

| Strain. | Sex. | Earliest tumour (months). | Effective number of mice. | Tumour mice. No. | Tumour mice. Percentage. | Tumour mice. Percentage (both sexes). | Tumour mice. Age at death (months). Range. | Tumour mice. Age at death (months). Average. | Tumour mice. Age at death (months). Average (both sexes). | Non-tumour mice. Age at death (months). Range. | Non-tumour mice. Age at death (months). Average. | Non-tumour mice. Age at death (months). Average (both sexes). |
|---|---|---|---|---|---|---|---|---|---|---|---|---|
| NBT | F | 9·0 | 240 | 28 | 11·7 | 7·7 | 12·0–23·0 | 16·4 | 15·8 | 9·0–24·0 | 14·2 | 13·3 |
| | M | | 214 | 7 | 3·3 | | 9·0–17·0 | 14·0 | | 9·0–22·0 | 12·3 | |
| CBA | F | 22·0 | 172 | 4 | 2·3 | 1·4 | 22·0–28·0 | 25·3 | | 22·0–44·0 | 26·7 | 26·1 |
| | M | | 109 | 0 | 0·0 | | — — | — | | 22·0–34·0 | 25·3 | |
| M/NBT | F | 10·5 | 22 | 17 | 77·3 | 60·7 | 10·5–17·5 | 14·7 | | 11·0–16·0 | 13·8 | 12·5 |
| | M | | 6 | 0 | 0·0 | | — — | — | | 10·5–12·5 | 11·4 | |
| M/CBA | F | 18·0 | 4 | 4 | 100·0 | 45·5 | 20·0–27·0 | 23·0 | 22·0 | — — | — | |
| | M | | 7 | 1 | 14·3 | | 18·0 — | 18·0 | | 20·0–26·0 | 22·3 | |
| M/NBT $F_1$ | F | 11·0 | 68 | 20 | 29·4 | 22·4 | 11·0–22·5 | 17·7 | 16·4 | 11·0–21·0 | 16·5 | 15·1 |
| | M | | 66 | 10 | 15·2 | | 11·5–19·0 | 14·2 | | 11·0–18·5 | 13·9 | |
| M/CBA $F_1$ | F | 20·0 | 162 | 7 | 4·3 | 3·7 | 20·0–30·0 | 27·0 | 25·1 | 20·0–34·0 | 26·1 | 24·7 |
| | M | | 109 | 3 | 2·8 | | 20·0–24·0 | 21·7 | | 20·0–25·0 | 22·8 | |

males in 214 had these tumours; as the males died 2 months earlier than the females, the sex-difference in tumour incidence, which was significant,[1]* could be due to the shorter life of the males. Non-tumorous mice of both sexes died on an average 2 months earlier than tumorous mice; the percentage tumour incidences of 11·7 (females) and 3·3 (males) may not therefore be a complete expression of the strain susceptibility.

In the CBA strain only 4 tumorous mice, all females, were seen. Non-tumorous males and females lived as long as, or longer than, the tumorous. The CBA strain therefore had a much lower degree of susceptibility than the NBT, the difference being significant,[2] and tumours were found much later than in the NBT.

2. *Induced tumours.*

Tumours were found in the M/NBT group in 17 of the 22 females living to 10 months and over, autopsied after December, 1946. Only 6 males fulfilled these requirements and none had tumours, possibly because none lived longer than one year. The non-tumorous females lived to about the same age as the tumorous. One tumour was a large spindle-celled sarcoma, the remainder were papillomata. The difference in tumour incidence between the sexes was significant,[3] as was the increase in incidence in the injected females compared with the controls,[4] in spite of the earlier deaths of the former. The control males had a significantly higher incidence[5] than the injected males (3·3 per cent compared with nil), but some of the former lived to a maximum of 22 months, whereas the maximum for the latter was 12·5 months (Table I).

The numbers of M/CBA mice surviving to tumour age (18 months) and autopsied after December 1946 were very small. All 4 females but only one of the 7

* For [1] and following numbers see Appendix.

males had tumours, a significant difference[6] and possibly a real one, since all the non-tumour males lived beyond tumour age. Two females had large epitheliomata of the forestomach, the remaining tumours being papillomata. The tumour incidence was significantly higher in the injected females (100 per cent) than in the controls,[7] but in the injected males (14·3 per cent) the increase was not significant owing to the small numbers involved.

3. *Uninjected M/NBT $F_1$ and M/CBA $F_1$.*

These mice were the generation raised from inbreeding the injected pure strain animals. As in the pure strain controls, all the tumours were papillomata.

In M/NBT $F_1$ twice as many females as males were tumorous, a significant difference,[8] but one which could again be explained by the earlier deaths of the males. Although the non-tumorous males lived about as long as the tumour males, they died 2·6 months earlier than the non-tumorous females (Table I). Compared with the control NBT group, the $F_1$ tumour incidences of 29·4 per cent (females) and 15·2 per cent (males) were significantly higher.[9] This supports the earlier statement that the control tumour incidences might have been higher if the mice had lived longer, for the M/NBT $F_1$ non-tumour females lived on the average nearly as long as the $F_1$ tumour females and 2 months longer than the control NBT non-tumour females, thus giving greater opportunity for a true expression of the strain susceptibility. The $F_1$ non-tumour males also lived longer than the control non-tumour males, by 1·6 months.

Comparing the tumour incidences in the $F_1$ and the M/NBT groups, the incidence in the $F_1$ females was significantly less[10] although the $F_1$ females lived much longer ; the incidence in the $F_1$ males should be compared not with that in the injected males (which were very few in number and died young) but with that in the injected females ; these lived to the same age, yet the $F_1$ male tumour incidence was very much less.

In the M/CBA $F_1$ group, although there were fewer tumours in the males (again due to their earlier deaths), the difference in tumour incidence between the sexes was not significant and the incidences were also in agreement with those of the CBA controls, males and females. Compared with the injected M/CBA group, the $F_1$ females had a significantly lower tumour incidence,[11] but the incidence in the males, although less in $F_1$, was not significantly so on account of the small number of M/CBA males.

Since the apparent sex differences in tumour incidence were most probably due to different survival times, the average incidences for both sexes combined in each group were compared and the following results obtained. Tumour incidences in the NBT and uninjected M/NBT $F_1$ groups were higher than in the CBA and uninjected M/CBA $F_1$ groups, but in the injected mice of both strains they were the same, the carcinogen having obliterated the strain difference. Tumours were much more frequent in the two injected groups than in the controls and malignant tumours were found after injection but not in the controls. The uninjected descendants of each M/NBT and M/CBA group had lower incidences than their injected parents, but, whereas M/CBA $F_1$ had the same incidence as the CBA controls, the tumour incidence in M/NBT $F_1$ was higher than that in the NBT controls because the $F_1$ mice lived longer.

4. *Multiple papillomata.*

The effect of the methylcholanthrene was seen not only in the increased incidence of tumorous mice but also in the increased number (and size) of tumours per mouse, as shown in Table II.

*NBT strain.*—In the control NBT group the majority of tumorous mice each had only one small nodule in the forestomach, but 4 (11·4 per cent) females had 2 nodules each. Of the 17 tumorous M/NBT mice 7 (41·2 per cent) had 2 or more

TABLE II.—*The Incidence of Multiple Papillomata and of Gross Malignant Tumours of the Forestomach in Control and Injected Tumorous Mice Belonging to the Inbred NBT and CBA Parent Strains and the First Two Generations of Reciprocal Hybrids.*

| Strain. | Generation. | Sex. | Number of tumour mice. | Mice with multiple papillomata. No. | Mice with multiple papillomata. Percentage. | | Maximum counted number of papillomata per stomach. | Number of mice with gross malignant tumours. |
|---|---|---|---|---|---|---|---|---|
| NBT | — | F | 28 | 4 | 14·3 | 11·4 | 2 | 0 |
| | | M | 7 | 0 | 0·0 | | 1 | 0 |
| M/NBT | — | F | 17 | 7 | 41·2 | 41·2 | 3* | 1 |
| | | M | 0 | 0 | 0·0 | | 0 | 0 |
| M/NBT | $F_1$ | F | 20 | 8 | 40·0 | 40·0 | 6* | 0 |
| | | M | 10 | 4 | 40·0 | | 3 | 0 |
| CBA | — | F | 4 | 0 | 0·0 | 0·0 | 1 | 0 |
| | | M | 0 | 0 | 0·0 | | 0 | 0 |
| M/CBA | — | F | 4 | 0 | 0·0 | 0·0 | 1 | 2 |
| | | M | 1 | 0 | 0·0 | | 1 | 0 |
| M/CBA | $F_1$ | F | 7 | Not stated | — | | — | 0 |
| | | M | 3 | in records | — | | — | 0 |
| NC | $F_1$ | F | 10 | 3 | 30·0 | 23·1 | 3 | 1 |
| | | M | 3 | 0 | 0·0 | | 1 | 0 |
| NC | $F_2$ | F | 13 | 2 | 15·4 | 7·4 | 3* | 0 |
| | | M | 14 | 0 | 0·0 | | 1 | 1 |
| CN | $F_1$ | F | 10 | 2 | 20·0 | 20·0 | 2 | 0 |
| | | M | 5 | 1 | 20·0 | | 2 | 0 |
| CN | $F_2$ | F | 9 | 2 | 22·2 | 11·8 | 2 | 0 |
| | | M | 8 | 0 | 0·0 | | 1 | 0 |
| MNC | $F_1$ | F | 10 | 5 | 50·0 | 56·5 | 2* | 2 |
| | | M | 13 | 8 | 61·5 | | 6 | 1 |
| MNC | $F_2$ | F | 29 | 14 | 48·3 | 37·8 | 4* | 3 |
| | | M | 16 | 3 | 18·8 | | 4 | 2 |
| MCN | $F_1$ | F | 21 | 16 | 76·2 | 71·4 | 4* | 4 |
| | | M | 14 | 9 | 64·3 | | 6* | 0 |
| MCN | $F_2$ | F | 35 | 21 | 60·0 | 55·3 | 7* | 3 |
| | | M | 12 | 5 | 41·7 | | 3 | 1 |

* = Large number, uncounted, recorded as "multiple."

nodules each, one mouse having 3 and another so many that they were not counted. This increase is statistically significant.[12] There were as many tumour mice with multiple papillomata in the M/NBT $F_1$ group as in the injected group, the maximum number counted in one mouse being 6, while another had so many that they were recorded simply as " multiple ". Although the $F_1$ mice were the longest-lived, this does not seem to be sufficient explanation of this result.

*CBA strain*—In the various CBA and M/CBA groups all the recorded occurrences were of solitary nodules. This is a further proof of the greater susceptibility of the NBT strain towards the formation of forestomach papillomata.

### B. *Hybrid Strains—First Two Generations.*

1. *Spontaneous tumours in $F_1$ and $F_2$.*

In these two generations the numbers of mice available for analysis were considerably reduced by the necessity of omitting all dying before December 1946. Data for the incidence of forestomach tumours are given in Table III.

In each of the four groups, NC $F_1$, NC $F_2$, CN $F_1$ and CN $F_2$, the tumour incidences in the two sexes were in agreement. Where they appeared to differ widely, as in NC $F_1$ and CN $F_1$, the numbers were small, the males died 4 or 5 months earlier than the females, but the differences were not statistically significant. Where the males lived as long as the females, as in NC $F_2$ and CN $F_2$, the incidence in the males was slightly higher than in the females, but not significantly so. In all four groups the non-tumour mice lived as long as the tumour mice and the average age at death was about 2 years. Comparing the various groups the tum-

TABLE III.—*The Incidence of Forestomach Tumours in Control and Injected Mice of the First Two Generations of Reciprocal Hybrids.*

| Strain and generation. | Sex. | Earliest tumour (months). | Effective number of mice. | Tumour mice. No. | Percentage. | | Age at death (months). Range. | Average. | | Non-tumour mice. Age at death (months). Range. | Average. | |
|---|---|---|---|---|---|---|---|---|---|---|---|---|
| NC $F_1$ | F | 16·5 | 57 | 10 | 17·5 | 13·7 | 16·5–35·0 | 27·8 | 26·4 | 18·0–35·0 | 27·0 | 25·2 |
| | M | | 38 | 3 | 7·9 | | 21·5–23·0 | 22·0 | | 16·5–31·5 | 22·8 | |
| NC $F_2$ | F | 16·5 | 103 | 13 | 12·6 | 13·2 | 19·0–29·0 | 24·5 | 23·7 | 16·5–31·5 | 24·1 | 23·6 |
| | M | | 101 | 14 | 13·9 | | 16·5–28·0 | 23·0 | | 16·5–31·5 | 23·0 | |
| CN $F_1$ | F | 17·5 | 54 | 10 | 18·5 | 14·3 | 19·5–34·0 | 27·3 | 25·9 | 17·5–34·0 | 27·2 | 25·3 |
| | M | | 51 | 5 | 9·8 | | 17·5–28·0 | 23·2 | | 17·5–31·5 | 23·9 | |
| CN $F_2$ | F | 18·0 | 117 | 9 | 7·7 | 8·2 | 18·0–28·5 | 23·9 | 23·7 | 18·0–32·5 | 27·6 | 24·3 |
| | M | | 90 | 8 | 8·9 | | 20·5–25·5 | 23·5 | | 18·0–31·0 | 22·7 | |
| MNC $F_1$ | F | 9·0 | 17 | 10 | 58·8 | 57·5 | 14·0–26·0 | 18·8 | 18·5 | 9·0–31·0 | 15·9 | 16·4 |
| | M | | 23 | 13 | 56·5 | | 9·0–29·5 | 18·3 | | 10·5–32·0 | 16·7 | |
| MNC $F_2$ | F | 6·0 | 94 | 29 | 30·9 | 23·6 | 6·0–24·5 | 16·0 | 15·5 | 6·0–26·5 | 11·6 | 11·6 |
| | M | | 97 | 16 | 16·5 | | 8·5–22·5 | 14·5 | | 6·0–23·0 | 11·7 | |
| MCN $F_1$ | F | 8·0 | 27 | 21 | 77·8 | 63·6 | 9·0–24·0 | 18·2 | 18·5 | 8·0–28·5 | 20·0 | 16·8 |
| | M | | 28 | 14 | 50·0 | | 8·0–28·0 | 18·9 | | 8·5–24·0 | 15·4 | |
| MCN $F_2$ | F | 8·0 | 74 | 35 | 47·3 | 35·1 | 8·0–28·0 | 15·6 | 15·0 | 8·0–28·5 | 15·4 | 13·9 |
| | M | | 60 | 12 | 20·0 | | 9·0–18·5 | 13·5 | | 8·0–27·0 | 12·6 | |

our incidences were all found to be in agreement, NC $F_1$ with NC $F_2$, CN $F_1$ with CN $F_2$, NC $F_1$ with CN $F_1$, and NC $F_2$ with CN $F_2$, the differences between percentages being less than twice the standard errors in all cases.

The tumour incidences in NC $F_1$ and CN $F_1$ were compared with those of NBT and also of M/NBT $F_1$, the latter on the assumption that it more truly represented the pure strain incidence owing to longer life. In none of the cases were the differences significant. The incidence in the $F_1$ hybrids was, therefore, that of the parent NBT strain—there was no sex-difference and no difference according to the direction of the cross.

As the mice of the early hybrid generations were long-lived, their tumours were found much later than those in the NBT mice and in this respect they resembled the other parent strain, the CBA. Although the tumours were of slow growth and could therefore have been present for a long time before death, yet mice dying in the younger age-groups were tumour-free, and it is believed that tumour development actually took place much later in the hybrids, being a function of the normal length of life of the mice.

With the exception of one gross epithelioma in an NC $F_1$ female and one in an NC $F_2$ male, the spontaneous tumours in the four groups were all papillomata.

2. *Induced tumours in $F_1$ and $F_2$.*

Data for tumour incidence in these generations of injected mice are given in Table III. The incidences were all much higher in the injected groups than in the controls. There was no sex-difference in tumour incidence in MNC $F_1$, but in the other three groups the incidences were significantly less in the males than in the females.[13] In MCN $F_1$ and MCN $F_2$ the non-tumour males died on the average several months earlier than the females, and in MNC $F_2$ the non-tumour mice of both sexes died at a very early average age.

Comparing MNC $F_1$ with MNC $F_2$ and MCN $F_1$ with MCN $F_2$, the drop in tumour incidence in the $F_2$ mice was significant in both cases,[14] and could be explained by the fact that the average age at death of injected $F_2$ mice was from 3 to nearly 5 months earlier than that of injected $F_1$ mice. There was no corresponding significant decline in the tumour incidence in the control $F_2$ mice, in which the decrease in the average age at death from $F_1$ to $F_2$ was much less.

Tumour incidences in MNC $F_1$ and MCN $F_1$ were in agreement, but the incidence of 23·6 per cent in MNC $F_2$ was significantly lower than that of 35·1 per cent in MCN $F_2$. Separate analysis showed that the difference lay in the females,[15] the incidences in the males being in agreement. The non-tumorous MNC $F_2$ females died 4 months earlier than the non-tumorous MCN $F_2$ females and also 4 months earlier than the tumorous MNC $F_2$ females ; the tumour susceptibility of the MNC $F_2$ females was therefore probably not fully expressed.

Comparisons were made between the tumour incidences in the injected $F_1$ and $F_2$ hybrids and in the corresponding groups of controls. In every case the incidence in the injected mice was significantly higher.[16] As shown in Table III, tumours were found in injected mice from 7·5 to 8·5 months earlier than in the controls.

While the majority of tumours were papillomata, some of which showed signs of early malignancy, a number of large malignant growths were found in the injected mice (Table II). Thus there were 3 epitheliomata in MNC $F_1$ and 5 (one consisting of mixed epithelioma and sarcoma elements) in MNC $F_2$ ; the 4

gross tumours in MCN $F_1$ included one pure sarcoma, 1 mixed epithelioma-sarcoma and 2 pure epitheliomata, and there were 4 epitheliomata in MCN $F_2$.

3. *Multiple papillomata.*

As shown in Table II, the number of tumour mice bearing multiple papillomata was much lower in the control hybrids than in the injected hybrids. Widely though the percentages with multiple nodules vary between NC $F_1$ and $F_2$ and CN $F_1$ and $F_2$, none of the differences is significant and the percentages do not differ significantly from the proportion of NBT tumour mice bearing multiple papillomata. One NC $F_1$ mouse had 3 nodules, one in NC $F_2$ had so many that they were not counted, and another in $F_2$ had 3 nodules.

The contrast in the injected hybrids was striking. Except in MNC $F_2$, the percentage of tumour mice bearing 2 or more nodules was over 50 per cent in each group. The maximum number counted in one stomach was 7, but many were recorded as " multiple". In spite of quite wide fluctuations, the proportions with multiple nodules agreed amongst themselves in the different groups of injected hybrids (the difference being always less than twice the standard error) and all were significantly greater than in the corresponding control groups[17] (the difference being always greater than twice the standard error).

As stated in a previous communication (Miller and Pybus, 1954*b*), there was a possibility that some mice of the control $F_1$ groups (NC $F_1$ and CN $F_1$) might have become contaminated with methylcholanthrene either by contact with, or by licking injected mice of the same litters kept in the same boxes ; the lung tumour incidences in these two groups were as high as in the injected $F_1$ mice. From the present data there is no positive evidence either from actual tumour incidence or from the percentages with multiple papillomata that such contamination, if it occurred, was sufficient to cause an increased incidence of forestomach tumours. The only possible evidence was, first, the case of the NC $F_1$ mouse bearing 3 papillomata (but one NC $F_2$ mouse had 3, and another " multiple ", nodules and there was no possibility that any control $F_2$ animal had come in contact with the carcinogen) and, second, the finding of a large malignant epithelioma in an NC $F_1$ mouse (but again an $F_2$ animal was found to have a large malignant epithelioma, presumably spontaneous).

### C. *Hybrid Strains—All Generations.*

1. *Spontaneous tumours, NC and CN groups.*

The data for spontaneous tumours in each generation of the NC and CN groups are given in Table IV, the figures for $F_1$ and $F_2$ being repeated from Table III for convenience of comparison. Comparing the same sexes, the total incidences for the 12 generations of 10·6 per cent for NC females and 8·5 per cent for CN females were in statistical agreement, as were the incidences of 5·5 per cent in both male groups.

In the NC group, although the incidences in the two sexes differed greatly in most generations, none of the differences was significant, but the totals of 10·6 per cent in females and 5·5 per cent in males in the 12 generations did differ significantly[18] (difference = 5·1, twice standard error = 3·3). In the CN group, the sex-differences in $F_9$ and $F_{11}$ only were significant[19] (in $F_9$, difference = 10·0, twice standard error = 9·5 ; in $F_{11}$, difference = 7·1, twice standard error = 6·9),

TABLE IV.—*The Incidence of Spontaneous Tumours of the Forestomach in Each Generation of Control NC and CN Hybrids.*

| Strain and generation. | Sex. | Effective number of mice. | Tumour mice. No. | Tumour mice. Percentage. | (F + M) | Tumour mice. Age at death (months). Range. | Tumour mice. Age at death (months). Average. | (F + M) | Non-tumour mice. Age at death (months). Range. | Non-tumour mice. Age at death (months). Average. | (F + M) |
|---|---|---|---|---|---|---|---|---|---|---|---|
| NC | | | | | | | | | | | |
| $F_1$ | F | 57 | 10 | 17·5 | 13·7 | 16·5–35·0 | 27·8 | 26·4 | 18·0–35·0 | 27·0 | 25·2 |
| | M | 38 | 3 | 7·9 | | 21·5–23·0 | 22·0 | | 16·5–31·5 | 22·8 | |
| $F_2$ | F | 103 | 13 | 12·6 | 13·2 | 19·0–29·0 | 24·5 | 23·7 | 16·5–31·5 | 24·1 | 23·6 |
| | M | 101 | 14 | 13·9 | | 16·5–28·0 | 23·0 | | 16·5–31·5 | 23·0 | |
| $F_3$ | F | 54 | 9 | 16·7 | 10·3 | 13·0–29·5 | 23·6 | 23·2 | 11·0–30·0 | 20·9 | 18·4 |
| | M | 43 | 1 | 2·3 | | 19·5 — | 19·5 | | 8·0–31·0 | 15·9 | |
| $F_4$ | F | 60 | 4 | 6·7 | 8·5 | 19·5–27·0 | 24·6 | 19·6 | 9·0–29·0 | 22·5 | 20·3 |
| | M | 46 | 5 | 10·9 | | 12·5–19·0 | 15·6 | | 8·0–25·0 | 17·2 | |
| $F_5$ | F | 40 | 3 | 7·5 | 3·5 | 14·0–26·0 | 20·0 | 20·0 | 9·0–27·0 | 21·7 | 19·3 |
| | M | 46 | 0 | 0·0 | | — — | — | | 9·0–24·0 | 17·4 | |
| $F_6$ | F | 45 | 3 | 6·7 | 4·2 | 16·5–21·5 | 21·6 | 21·6 | 8·0–25·0 | 19·1 | 17·5 |
| | M | 27 | 0 | 0·0 | | — — | — | | 8·0–21·0 | 14·8 | |
| $F_7$ | F | 22 | 1 | 4·5 | 4·4 | 22·0 — | 22·0 | 16·3 | 16·0–28·0 | 20·1 | 17·4 |
| | M | 46 | 2 | 4·4 | | 9·0–18·0 | 13·5 | | 8·0–23·0 | 16·2 | |
| $F_8$ | F | 48 | 7 | 14·6 | 8·7 | 14·5–26·5 | 21·2 | 20·7 | 9·0–27·0 | 19·5 | 15·5 |
| | M | 56 | 2 | 3·6 | | 16·0–21·5 | 18·8 | | 8·0–23·0 | 12·4 | |
| $F_9$ | F | 36 | 4 | 11·1 | 6·9 | 14·0–21·0 | 17·4 | 15·5 | 9·0–22·0 | 17·3 | 15·3 |
| | M | 36 | 1 | 2·8 | | 8·0 — | 8·0 | | 8·0–20·0 | 13·5 | |
| $F_{10}$ | F | 31 | 1 | 3·2 | 1·5 | 8·0 — | 8·0 | 8·0 | 8·0–29·0 | 17·7 | 14·6 |
| | M | 35 | 0 | 0·0 | | — — | — | | 8·0–19·0 | 11·7 | |
| $F_{11}$ | F | 23 | 1 | 4·4 | 3·1 | 11·0 — | 11·0 | 11·0 | 8·0–24·0 | 17·0 | 14·9 |
| | M | 9 | 0 | 0·0 | | — — | — | | 8·0–15·0 | 10·9 | |
| $F_{12}$ | F | 28 | 2 | 7·1 | 3·7 | 12·5–14·0 | 13·3 | 13·3 | 9·0–23·0 | 14·8 | 14·0 |
| | M | 26 | 0 | 0·0 | | — — | — | | 8·0–20·0 | 13·3 | |
| $F_1$–$F_{12}$ | F | 547 | 58 | 10·6 | 8·1 | 8·0–35·0 | 22·7 | 21·8 | 8·0–35·0 | 21·1 | 18·9 |
| | M | 509 | 28 | 5·5 | | 8·0–28·0 | 19·9 | | 8·0–31·5 | 16·8 | |
| CN | | | | | | | | | | | |
| $F_1$ | F | 54 | 10 | 18·5 | 14·3 | 19·5–34·0 | 27·3 | 25·9 | 17·5–34·0 | 27·2 | 25·3 |
| | M | 51 | 5 | 9·8 | | 17·5–28·0 | 23·2 | | 17·5–31·5 | 23·9 | |
| $F_2$ | F | 117 | 9 | 7·7 | 8·2 | 18·0–28·5 | 23·9 | 23·7 | 18·0–32·5 | 27·6 | 24·3 |
| | M | 90 | 8 | 8·9 | | 20·5–25·5 | 23·5 | | 18·0–31·0 | 22·7 | |
| $F_3$ | F | 40 | 7 | 17·5 | 14·3 | 10·0–28·5 | 22·5 | 20·6 | 10·0–32·0 | 23·2 | 20·6 |
| | M | 33 | 4 | 12·1 | | 12·5–23·0 | 17·4 | | 9·0–28·0 | 18·6 | |
| $F_4$ | F | 35 | 4 | 11·4 | 14·1 | 22·0–29·5 | 25·2 | 22·4 | 9·0–29·0 | 20·4 | 18·3 |
| | M | 36 | 6 | 16·7 | | 11·0–25·0 | 20·2 | | 8·0–28·0 | 16·1 | |
| $F_5$ | F | 28 | 3 | 10·7 | 5·9 | 8·0–26·5 | 19·0 | 19·0 | 8·0–33·0 | 22·4 | 19·4 |
| | M | 23 | 0 | 0·0 | | — — | — | | 8·0–23·0 | 16·2 | |
| $F_6$ | F | 25 | 2 | 8·0 | 5·3 | 21·0–26·5 | 23·8 | 23·8 | 11·0–27·0 | 19·9 | 16·6 |
| | M | 13 | 0 | 0·0 | | — — | — | | 8·0–14·0 | 10·8 | |

TABLE IV—*cont.*

| | | | Tumour mice. | | | | | | Non-tumour mice. Age at death (months). | | |
|---|---|---|---|---|---|---|---|---|---|---|---|
| | | | | | | Age at death (months). | | | | | |
| Strain and generation. | Sex. | Effective number of mice. | No. | Percentage. | | Range. | Average. | | Range. | Average. | |
| $F_7$ | F | 24 | 1 | 4·2 | 1·9 | 20·0 — | 20·0 | 20·0 | 8·0–30·0 | 17·6 | 14·9 |
| | M | 29 | 0 | 0·0 | | — — | — | | 8·0–24·0 | 12·7 | |
| $F_8$ | F | 38 | 4 | 10·5 | 7·8 | 20·5–27·5 | 24·6 | 19·9 | 10·0–28·0 | 20·6 | 16·3 |
| | M | 39 | 2 | 5·1 | | 9·5–11·5 | 10·5 | | 8·0–23·0 | 12·3 | |
| $F_9$ | F | 40 | 4 | 10·0 | 6·7 | 25·0–30·0 | 26·6 | 26·6 | 9·0–32·0 | 20·3 | 16·8 |
| | M | 20 | 0 | 0·0 | | — — | — | | 8·0–14·0 | 10·7 | |
| $F_{10}$ | F | 53 | 2 | 3·8 | 3·4 | 16·0–22·0 | 19·0 | 17·2 | 8·0–28·0 | 17·9 | 15·6 |
| | M | 36 | 1 | 2·8 | | 13·5 — | 13·5 | | 8·0–18·0 | 12·4 | |
| $F_{11}$ | F | 56 | 4 | 7·1 | 4·8 | 10·0–20·0 | 13·8 | 13·8 | 8·0–24·0 | 15·6 | 13·5 |
| | M | 28 | 0 | 0·0 | | — — | — | | 8·0–15·0 | 9·5 | |
| $F_{12}$ | F | 116 | 3 | 2·6 | 2·4 | 18·5–24·5 | 21·7 | 18·6 | 8·0–29·0 | 16·3 | 14·8 |
| | M | 131 | 3 | 2·3 | | 13·0–27·0 | 15·5 | | 8·0–19·0 | 13·4 | |
| $F_1$–$F_{12}$ | F | 620 | 53 | 8·5 | 7·1 | 8·0–34·0 | 23·3 | 22·1 | 8·0–34·0 | 19·3 | 17·3 |
| | M | 529 | 29 | 5·5 | | 9·5–28·0 | 19·9 | | 8·0–31·5 | 15·0 | |

and the difference between the totals for the 12 generations of 8·5 per cent in females and 5·5 per cent in males was barely significant (difference = 3·0, twice standard error = 2·99). In both groups the males died several months earlier than the females, and this is believed to account for the difference in tumour incidences in the two sexes.

In the later generations of both groups there was a tendency towards earlier death in both sexes and this is reflected in the trend towards lower tumour incidences in later generations, this trend being noticeable in spite of the wide fluctuations from generation to generation. While there might be a genetical basis for the diminishing incidences, it was not possible to discern this in the case of a tumour essentially of old age and with such a low incidence in younger mice that often there was only a solitary occurrence, or even none, in a small generation in which many mice died young.

Spontaneous gross malignant tumours were rare, none being seen in the CN group, and (in addition to those mentioned earlier) only 1 in an NC $F_3$ female and 2 in NC $F_8$ females, all squamous epitheliomata, giving a total in NC of 4 gross tumours in females and 1 in a male.

Multiple papillomata. All the other spontaneous tumours in these groups were papillomata, the great majority being solitary. In NC ($F_3$ to $F_{12}$) only 2 out of 5 $F_3$ tumour males and 1 out of 7 $F_8$ tumour females each had 2 papillomata ; including $F_1$ and $F_2$ (Table II) this gave a total of 6 cases of multiple nodules in 58 tumour females (10·4 per cent) and 2 in 28 tumour males (7·1 per cent). This difference was not significant. In the control CN mice ($F_3$ to $F_{12}$) 1 $F_4$ female had 2 papillomata, the remainder being solitary. With $F_1$ and $F_2$ (Table II) this gave a total of 5 cases of multiple nodules in 53 tumour females (9·4 per cent) and 1 in 29 males (3·4 per cent), again an insignificant difference. The incidences of multiple nodules in the two control groups were in agreement for both males and females.

2. *Induced tumours, MNC and MCN groups.*

Table V presents the data for the tumour incidence in each generation of the injected hybrids, the figures for $F_1$ and $F_2$ being repeated from Table III for comparison. There was wide variation in tumour incidence between the sexes. In MNC $F_1$, $F_3$, $F_6$ and $F_9$ this difference was not significant, but in the remaining generations and in the totals for all 10 generations in this group the differences were significant.[20] In the MCN group the sex-difference in tumour incidence was significant in $F_1$, $F_2$ and $F_3$ and in the total for the 10 injected generations.[21] In both injected groups the non-tumour mice died young, their average age being several months less than the average tumour age, but the difference in age at death between males and females was much less constant than in the control NC and CN hybrids.

In both groups there was a decrease in tumour incidence in the later generations compared especially with $F_1$ and $F_2$, and this was paralleled by a decrease in the average survival age of the mice. The very low incidences in the last 5 generations of MCN were due to the early deaths, very few mice living to the age of 18 months.

The total tumour incidences in the two injected groups were in agreement, females with females (27·7 per cent in MNC, 27·2 per cent in MCN) and males with males (12·5 per cent in MNC, 14·1 per cent in MCN). There was also very little difference between the average ages at death of tumour mice in the two groups, and also of non-tumour mice.

Tumour incidences were significantly higher in the MNC and MCN hybrids than in the control NC and CN groups,[22] by as much as two to three times, and tumours were found much earlier in the injected groups—by about 8 months.

*Multiple papillomata.*—Table VI shows the incidence of multiple papillomata in the injected MNC and MCN generations. Nearly 50 per cent of the injected tumorous mice had 2 or more nodules each, and whereas in the controls (where less than 10 per cent of tumorous mice bore 2 or more nodules) the maximum number counted in one individual was 3, in the injected mice the maximum number counted was 9, and there were many cases recorded simply as " multiple ", the papillomata being so numerous.

The proportions of tumour-bearing injected mice in the two groups which had multiple papillomata were in agreement (48·8 per cent of 168 MNC tumour females and 46·0 per cent of 124 MCN tumour females ; 37·1 per cent of 89 MNC tumour males and 44·6 per cent of 65 MCN tumour males) and there was no significant sex-difference.

There were 26 cases of gross epitheliomata and sarcomata in the MNC mice and 16 cases in the MCN mice.

3. *The uninjected MNC and MCN groups.*

Table VII gives the incidence of spontaneous tumours in these descendants of injected mice. In the uninjected MNC group the differences between the sexes were not significant except in $F_{12}$ (a large generation) and in the totals for $F_5$ to $F_{12}$.[23] Tumour incidence fluctuated from generation to generation, and the totals

TABLE V.—*The Incidence of Forestomach Tumours in Each Generation of Methylcholanthrene-injected MNC and MCN Hybrids.*

| Strain and generation. | Sex. | Effective number of mice. | Tumour mice. No. | Percentage. | | Age at death (months). Range. | Average. | | Non-tumour mice. Age at death (months). Range. | Average. | |
|---|---|---|---|---|---|---|---|---|---|---|---|
| MNC | | | | | | | | | | | |
| $F_1$ | F | 17 | 10 | 58·8 | 57·5 | 14·0–26·0 | 18·8 | 18·5 | 9·0–31·0 | 15·9 | 16·4 |
| | M | 23 | 13 | 56·5 | | 9·0–29·5 | 18·3 | | 10·5–32·0 | 16·7 | |
| $F_2$ | F | 94 | 29 | 30·9 | 23·6 | 6·0–24·5 | 16·0 | 15·5 | 6·0–26·5 | 11·6 | 11·6 |
| | M | 97 | 16 | 16·5 | | 8·5–22·5 | 14·5 | | 6·0–23·0 | 11·7 | |
| $F_3$ | F | 33 | 9 | 27·3 | 21·7 | 5·0–23·0 | 13·8 | 12·0 | 5·0–17·0 | 9·5 | 8·6 |
| | M | 36 | 6 | 16·7 | | 6·5–11·5 | 9·3 | | 5·0–13·0 | 7·8 | |
| $F_4$ | F | 107 | 50 | 46·7 | 36·9 | 5·5–26·5 | 13·4 | 12·0 | 4·0–29·0 | 9·4 | 8·6 |
| | M | 110 | 30 | 27·3 | | 4·0–19·5 | 9·7 | | 4·0–24·0 | 7·9 | |
| $F_5$ | F | 60 | 23 | 36·3 | 20·3 | 5·0–24·5 | 10·4 | 10·1 | 4·0–23·5 | 7·9 | 7·6 |
| | M | 88 | 7 | 8·0 | | 5·0–20·5 | 9·2 | | 4·0–18·0 | 7·4 | |
| $F_6$ | F | 49 | 9 | 18·4 | 15·0 | 6·0–23·5 | 11·4 | 10·9 | 4·0–23·5 | 8·8 | 7·7 |
| | M | 51 | 6 | 11·8 | | 7·0–16·5 | 10·1 | | 4·0–17·0 | 7·4 | |
| $F_7$ | F | 84 | 14 | 16·7 | 9·4 | 7·0–27·0 | 16·4 | 15·6 | 4·0–28·0 | 9·0 | 8·7 |
| | M | 96 | 3 | 3·1 | | 8·5–16·5 | 11·5 | | 4·0–20·0 | 8·5 | |
| $F_8$ | F | 31 | 8 | 25·8 | 12·4 | 5·0–25·0 | 12·8 | 12·9 | 4·0–24·0 | 10·0 | 8·2 |
| | M | 50 | 2 | 4·0 | | 6·0–20·0 | 13·0 | | 5·0–17·0 | 7·4 | |
| $F_9$ | F | 62 | 5 | 8·1 | 5·0 | 14·5–22·0 | 18·4 | 16·0 | 4·0–23·0 | 8·7 | 9·1 |
| | M | 79 | 2 | 2·5 | | 6·5–13·5 | 10·0 | | 4·0–22·0 | 9·4 | |
| $F_{10}$ | F | 69 | 11 | 15·9 | 10·1 | 11·5–27·0 | 17·6 | 17·6 | 4·0–22·0 | 7·9 | 8·9 |
| | M | 80 | 4 | 5·0 | | 16·0–19·0 | 17·6 | | 4·0–25·0 | 9·6 | |
| $F_1$–$F_{10}$ | F | 606 | 168 | 27·7 | 19·5 | 5·0–27·0 | 14·3 | 13·6 | 4·0–31·0 | 9·3 | 9·1 |
| | M | 710 | 89 | 12·5 | | 4·0–29·5 | 12·3 | | 4·0–32·0 | 8·9 | |
| MCN | | | | | | | | | | | |
| $F_1$ | F | 27 | 21 | 77·8 | 63·6 | 9·0–24·0 | 18·2 | 18·5 | 8·0–28·5 | 20·0 | 16·8 |
| | M | 28 | 14 | 50·0 | | 8·0–28·0 | 18·9 | | 8·5–24·0 | 15·4 | |
| $F_2$ | F | 74 | 35 | 47·3 | 35·1 | 8·0–28·0 | 15·6 | 15·0 | 8·0–28·5 | 15·4 | 13·9 |
| | M | 60 | 12 | 20·0 | | 9·0–18·5 | 13·5 | | 8·0–27·0 | 12·6 | |
| $F_3$ | F | 45 | 23 | 51·1 | 31·9 | 5·5–27·0 | 17·6 | 16·8 | 6·0–26·0 | 12·4 | 11·9 |
| | M | 46 | 6 | 13·0 | | 9·0–19·5 | 13·5 | | 5·0–28·0 | 11·7 | |
| $F_4$ | F | 59 | 18 | 30·5 | 29·1 | 5·5–21·5 | 10·5 | 10·3 | 4·0–26·0 | 10·4 | 9·4 |
| | M | 58 | 16 | 27·6 | | 6·0–20·0 | 10·1 | | 4·0–24·0 | 8·5 | |
| $F_5$ | F | 58 | 18 | 31·0 | 25·0 | 7·5–24·5 | 13·0 | 12·0 | 4·0–23·0 | 9·7 | 9·7 |
| | M | 66 | 13 | 19·7 | | 4·0–19·5 | 10·5 | | 4·0–26·0 | 9·7 | |
| $F_6$ | F | 12 | 0 | 0·0 | 0·0 | — — | — | | 4·0–20·0 | 12·3 | 8·7 |
| | M | 13 | 0 | 0·0 | | — — | — | | 4·0– 8·0 | 5·5 | |
| $F_7$ | F | 25 | 0 | 0·0 | 0·0 | — — | — | | 5·0–17·0 | 9·3 | 8·9 |
| | M | 25 | 0 | 0·0 | | — — | — | | 4·0–16·0 | 8·4 | |
| $F_8$ | F | 53 | 4 | 7·6 | 4·9 | 7·0–16·5 | 12·1 | 11·2 | 4·0–18·0 | 8·2 | 8·0 |
| | M | 70 | 2 | 2·9 | | 7·5–11·5 | 9·5 | | 4·0–17·0 | 7·9 | |
| $F_9$ | F | 39 | 2 | 5·1 | 2·4 | 12·0–18·5 | 15·3 | 15·3 | 4·0–17·0 | 9·4 | 8·3 |
| | M | 44 | 0 | 0·0 | | — — | — | | 4·0–16·0 | 7·4 | |
| $F_{10}$ | F | 64 | 3 | 4·7 | 4·4 | 12·5–15·0 | 14·2 | 12·9 | 4·0–17·0 | 8·6 | 8·6 |
| | M | 50 | 2 | 4·0 | | 9·5–12·5 | 11·0 | | 4·0–15·0 | 8·6 | |
| $F_1$–$F_{10}$ | F | 456 | 124 | 27·2 | 20·6 | 5·5–28·0 | 15·1 | 14·4 | 4·0–28·5 | 10·4 | 9·9 |
| | M | 460 | 65 | 14·1 | | 4·0–28·0 | 13·0 | | 4·0–28·0 | 9·4 | |

TABLE VI.—*Incidences of Multiple Papillomata and of Gross Malignant Tumours of the Forestomach in Each Generation of Methylcholanthrene-injected MNC and MCN Hybrids.*

| Strain and generation. | Sex. | Number of tumour mice. | Mice with multiple papillomata. No. | Percentage. | | Maximum counted number of papillomata per stomach. | Number of mice with gross malignant tumours. |
|---|---|---|---|---|---|---|---|
| $F_1$ | F | 10 | 5 | 50·0 | 56·5 | 2* | 2 |
| | M | 13 | 8 | 61·5 | | 6 | 1 |
| $F_2$ | F | 29 | 14 | 48·3 | 37·8 | 4* | 3 |
| | M | 16 | 3 | 18·8 | | 4 | 2 |
| $F_3$ | F | 9 | 8 | 88·9 | 73·3 | 5 | 0 |
| | M | 6 | 3 | 50·0 | | 2* | 0 |
| $F_4$ | F | 50 | 30 | 60·0 | 57·5 | 9* | 7 |
| | M | 30 | 16 | 53·3 | | 3* | 4 |
| $F_5$ | F | 23 | 9 | 39·1 | 36·7 | 5 | 1 |
| | M | 7 | 2 | 28·6 | | 2 | 0 |
| $F_6$ | F | 9 | 6 | 66·7 | 40·0 | 4 | 0 |
| | M | 6 | 0 | 0·0 | | 1 | 0 |
| $F_7$ | F | 14 | 4 | 28·6 | 23·5 | 3 | 2 |
| | M | 3 | 0 | 0·0 | | 1 | 0 |
| $F_8$ | F | 8 | 2 | 25·0 | 30·0 | 2 | 0 |
| | M | 2 | 1 | 50·0 | | 5 | 0 |
| $F_9$ | F | 5 | 1 | 20·0 | 14·3 | 2 | 1 |
| | M | 2 | 0 | 0·0 | | 1 | 1 |
| $F_{10}$ | F | 11 | 3 | 27·3 | 20·0 | 3 | 2 |
| | M | 4 | 0 | 0·0 | | 1 | 0 |
| $F_1$–$F_{10}$ | F | 168 | 82 | 48·8 | 44·7 | 9* | 18 |
| | M | 89 | 33 | 37·1 | | 6* | 8 |
| MCN | | | | | | | |
| $F_1$ | F | 21 | 16 | 76·2 | 71·4 | 4* | 4 |
| | M | 14 | 9 | 64·3 | | 6* | 0 |
| $F_2$ | F | 35 | 21 | 60·0 | 55·3 | 7* | 3 |
| | M | 12 | 5 | 41·7 | | 3 | 1 |
| $F_3$ | F | 23 | 6 | 26·1 | 27·6 | 4* | 6 |
| | M | 6 | 2 | 33·3 | | 4 | 1 |
| $F_4$ | F | 18 | 8 | 44·4 | 50·0 | 5 | 0 |
| | M | 16 | 9 | 56·3 | | 5* | 1 |
| $F_5$ | F | 18 | 6 | 33·3 | 32·3 | 4 | 0 |
| | M | 13 | 4 | 30·8 | | 6 | 0 |
| $F_6$ | F | 0 | 0 | 0·0 | 0·0 | 0 | 0 |
| | M | 0 | 0 | 0·0 | | 0 | 0 |
| $F_7$ | F | 0 | 0 | 0·0 | 0·0 | 0 | 0 |
| | M | 0 | 0 | 0·0 | | 0 | 0 |
| $F_8$ | F | 4 | 0 | 0·0 | 0·0 | 1 | 0 |
| | M | 2 | 0 | 0·0 | | 1 | 0 |

TABLE VI—*cont.*

| Strain and generation. | Sex. | Number of tumour mice. | Mice with multiple papillomata. No. | Mice with multiple papillomata. Percentage. | | Maximum counted number of papillomata per stomach. | Number of mice with gross malignant tumours. |
|---|---|---|---|---|---|---|---|
| MNC | | | | | | | |
| $F_9$ | F | 2 | 0 | 0·0 | 0·0 | 1 | 0 |
| | M | 0 | 0 | 0·0 | | 0 | 0 |
| $F_{10}$ | F | 3 | 0 | 0·0 | 0·0 | 1 | 0 |
| | M | 2 | 0 | 0·0 | | 1 | 0 |
| $F_1$–$F_{10}$ | F | 124 | 57 | 46·0 | 45·5 | 7* | 13 |
| | M | 65 | 29 | 44·6 | | 6* | 3 |

* = Large number, uncounted, recorded as " multiple."

of 9·3 per cent for females and 4·4 per cent for males were in agreement with the control NC values of 10·6 per cent and 5·5 per cent respectively (Table IV). The ages at death of the tumorous and non-tumorous uninjected MNC mice were also comparable with the control values.

The uninjected MCN mice died young and the tumour incidences (1·9 per cent in females and 1·5 per cent in males) were low. The sex-differences were nowhere significant. The tumour incidences were significantly less than those in the uninjected MNC mice, and also significantly less than those in the CN controls.[24]

There was no evidence that the increased incidence and earlier appearance of forestomach tumours in the injected mice were characteristics continued in their uninjected descendants. Especially in $F_{12}$ of the uninjected MNC hybrids, a very large generation and one that was preceded by 10 generations of injected mice and one of uninjected, the tumour incidences of 10·8 per cent in the females and 5·0 per cent in the males were in agreement with the total incidences of 10·6 per cent and 5·5 per cent respectively in the NC controls.

*Multiple papillomata.*—Table VIII shows the incidence of multiple papillomata in the 8 generations of uninjected MNC mice. The maximum individual number was 3 in an $F_{11}$ mouse. The total incidences of 7·7 per cent in 78 tumorous females and 2·1 per cent in 48 tumour males (not a significant difference) did not differ significantly from the corresponding values in the control NC hybrids. There were 6 cases of malignant epitheliomata in the uninjected MNC group, 4 of which were quite small tumours and none was so florid as those found in the injected mice.

Of the few tumorous mice in the uninjected MCN group none had multiple papillomata, but 2 malignant epitheliomata, both rather small, were found in $F_5$.

### D. *Malignant Tumours.*

Bagshaw and Strong (1950) found that malignant epidermoid carcinomata of the forestomach appeared earlier than benign papillomata. The ages at death of all mice in the present experiment bearing malignant forestomach tumours

TABLE VII.—*The Incidence of Forestomach Tumours in the Uninjected Offspring of Injected Hybrids.*

| Strain and generation. | Sex. | Effective number of mice. | Tumour mice. No. | Tumour mice. Percentage. | | Tumour mice. Age at death (months). Range. | Tumour mice. Age at death (months). Average. | | Non-tumour mice. Age at death (months). Range. | Non-tumour mice. Age at death (months). Average. | |
|---|---|---|---|---|---|---|---|---|---|---|---|
| MNC (uninj.) | | | | | | | | | | | |
| $F_5$ | F | 27 | 0 | 0·0 | 1·6 | — — | — | 16·5 | 6·0–27·0 | 18·8 | 16·9 |
| | M | 35 | 1 | 2·9 | | 16·5 — | 16·5 | | 7·0–29·0 | 15·5 | |
| $F_6$ | F | 100 | 14 | 14·0 | 9·9 | 19·0–28·0 | 23·4 | 22·7 | 6·0–29·0 | 20·9 | 19·4 |
| | M | 112 | 7 | 6·3 | | 15·5–23·5 | 21·3 | | 6·0–30·0 | 18·1 | |
| $F_7$ | F | 77 | 4 | 5·2 | 5·9 | 19·0–27·0 | 23·0 | 21·2 | 6·0–29·0 | 21·0 | 18·5 |
| | M | 77 | 5 | 6·5 | | 15·5–27·0 | 19·8 | | 6·0–26·0 | 15·9 | |
| $F_8$ | F | 42 | 3 | 7·2 | 4·5 | 24·0–27·0 | 26·0 | 25·8 | 9·0–28·0 | 21·5 | 17·7 |
| | M | 46 | 1 | 2·2 | | 25·0 — | 25·0 | | 6·0–26·0 | 14·3 | |
| $F_9$ | F | 34 | 2 | 5·9 | 2·6 | 20·0–25·5 | 22·8 | 22·8 | 6·0–28·0 | 20·2 | 18·3 |
| | M | 44 | 0 | 0·0 | | — — | — | | 8·0–26·0 | 16·8 | |
| $F_{10}$ | F | 80 | 9 | 11·3 | 8·3 | 15·0–26·5 | 20·9 | 21·1 | 7·0–28·0 | 19·9 | 18·6 |
| | M | 77 | 4 | 5·2 | | 18·5–26·0 | 21·6 | | 6·0–27·0 | 17·4 | |
| $F_{11}$ | F | 147 | 10 | 6·8 | 4·5 | 6·0–28·5 | 19·2 | 19·7 | 6·0–26·0 | 17·8 | 17·1 |
| | M | 209 | 6 | 2·9 | | 14·5–23·5 | 20·5 | | 6·0–25·0 | 16·6 | |
| $F_{12}$ | F | 334 | 36 | 10·8 | 7·5 | 12·0–28·5 | 22·3 | 21·2 | 6·0–28·0 | 19·6 | 18·7 |
| | M | 440 | 22 | 5·0 | | 14·5–23·5 | 19·5 | | 6·0–28·0 | 18·1 | |
| $F_5$–$F_{12}$ | F | 841 | 78 | 9·3 | 6·6 | 6·0–28·5 | 22·1 | 21·4 | 6·0–29·0 | 17·9 | 17·1 |
| | M | 1040 | 46 | 4·4 | | 14·5–27·0 | 20·2 | | 6·0–30·0 | 16·5 | |
| MCN (uninj.) | | | | | | | | | | | |
| $F_5$ | F | 38 | 1 | 2·6 | 4·6 | 23·5 — | 23·5 | 19·9 | 11·0–25·0 | 19·3 | 18·7 |
| | M | 49 | 3 | 6·1 | | 10·0–25·0 | 18·7 | | 11·0–25·0 | 18·3 | |
| $F_6$ | F | 3 | 0 | 0·0 | 0·0 | — — | — | — | 16·0–20·0 | 18·0 | 17·8 |
| | M | 2 | 0 | 0·0 | | — — | — | | 14·0–21·0 | 17·5 | |
| $F_7$ | F | 17 | 0 | 0·0 | 0·0 | — — | — | — | 10·0–18·0 | 14·8 | 14·4 |
| | M | 24 | 0 | 0·0 | | — — | — | | 12·0–17·0 | 14·2 | |
| $F_8$ | F | 31 | 1 | 3·2 | 1·5 | 16·0 — | 16·0 | 16·0 | 11·0–19·0 | 15·4 | 14·2 |
| | M | 38 | 0 | 0·0 | | — — | — | | 10·0–17·0 | 13·2 | |
| $F_9$ | F | 40 | 1 | 2·5 | 3·9 | 18·0 — | 18·0 | 16·2 | 11·0–21·0 | 16·3 | 15·6 |
| | M | 36 | 2 | 5·6 | | 14·5–16·0 | 15·3 | | 10·0–22·0 | 14·8 | |
| $F_{10}$ | F | 46 | 1 | 2·2 | 1·9 | 18·5 — | 18·5 | 18·5 | 10·0–19·0 | 14·6 | 13·9 |
| | M | 38 | 0 | 0·0 | | — — | — | | 10·0–20·0 | 13·1 | |
| $F_{11}$ | F | 90 | 2 | 2·2 | 1·1 | 14·0–15·5 | 14·8 | 14·8 | 10·0–24·0 | 13·7 | 13·2 |
| | M | 85 | 0 | 0·0 | | — — | — | | 10·0–21·0 | 12·8 | |
| $F_{12}$ | F | 114 | 1 | 0·9 | 0·8 | 17·0 — | 17·0 | 15·5 | 10·0–20·0 | 13·6 | 13·3 |
| | M | 125 | 1 | 0·8 | | 14·0 — | 14·0 | | 10·0–17·0 | 13·1 | |
| $F_5$–$F_{12}$ | F | 379 | 7 | 1·9 | 1·7 | 14·5–23·5 | 17·5 | 17·2 | 10·0–25·0 | 14·8 | 14·4 |
| | M | 397 | 6 | 1·5 | | 10·0–25·0 | 16·8 | | 10·0–25·0 | 13·9 | |

TABLE VIII.—*Incidences of Multiple Papillomata and of Gross Malignant Tumours of the Forestomach in Each Generation of Uninjected Descendants of Injected MNC Mice.*

| Strain and generation. | Sex. | Number of tumour mice. | Mice with multiple papillomata. No. | Percentage. | | Maximum counted number of papillomata per stomach. | Number of mice with gross malignant tumours |
|---|---|---|---|---|---|---|---|
| MNC (uninjected) | | | | | | | |
| $F_5$ | F | 0 | 0 | 0·0 | 0·0 | 0 | 0 |
| | M | 1 | 0 | 0·0 | | 1 | 0 |
| $F_6$ | F | 14 | 0 | 0·0 | 0·0 | 1 | 3 |
| | M | 7 | 0 | 0·0 | | 1 | 0 |
| $F_7$ | F | 4 | 0 | 0·0 | 11·1 | 1 | 0 |
| | M | 5 | 1 | 20·0 | | 2 | 0 |
| $F_8$ | F | 3 | 1 | 33·3 | 25·0 | 2 | 0 |
| | M | 1 | 0 | 0·0 | | 1 | 0 |
| $F_9$ | F | 2 | 0 | 0·0 | 0·0 | 1 | 0 |
| | M | 0 | 0 | 0·0 | | 0 | 0 |
| $F_{10}$ | F | 9 | 2 | 22·2 | 15·4 | 2 | 0 |
| | M | 4 | 0 | 0·0 | | 1 | 0 |
| $F_{11}$ | F | 10 | 1 | 10·0 | 6·3 | 3 | 1 |
| | M | 6 | 0 | 0·0 | | 1 | 0 |
| $F_{12}$ | F | 36 | 2 | 5·6 | 3·4 | 2 | 0 |
| | M | 22 | 0 | 0·0 | | 1 | 2 |
| $F_5$–$F_{12}$ | F | 78 | 6 | 7·7 | 5·6 | 3 | 4 |
| | M | 46 | 1 | 2·2 | | 2 | 2 |

are shown in Table IX, for comparison with the average ages of discovery of all forestomach tumours, benign and malignant, given in Tables I, IV, V and VII. With a few exceptions, the ages of discovery of spontaneous and induced malignant gross tumours were slightly greater than the corresponding average ages for all tumours. It must again be pointed out that " tumour age " in the present experiment was the age at which the tumour was discovered, that a tumour might have been present in the animal for some time before death, and that a gross malignant tumour could be the cause of death. Stewart and Lorenz (1949) make a sharp distinction between benign papillomata and gross carcinomata, stating that the former do not necessarily lead to the latter ; and in the present material there were instances where both types were present in the same stomach.

### DISCUSSION.

Very few references have been found in the literature to the presence of spontaneous benign papillomata of the forestomach in mice. Stewart and Andervont (1938) quote Bonné (1927) who found 2 mice with stomach papillomata in a total of 146 controls, and Stewart and Lorenz (1949) found solitary papillomata in control mice receiving mineral oil. Peacock, Beck and Chalmers (1953) saw no spontaneous papillomata in 86 control mice, but found 3 in 99 mice treated with

TABLE IX.—*The Ages at Death of Mice Bearing Malignant Forestomach Tumours.*

| Strain and generation. | Individual ages (months). Females. | Males. | Average ages. Females. | Males. | Group averages. Females. | Males. |
|---|---|---|---|---|---|---|
| M/NBT | 13·5 | — | 13·5 | — | 13·5 | — |
| M/CBA | 20·0, 27·0 | — | 23·5 | — | 23·5 | — |
| NC | | | | | | |
| $F_1$ | 29·0 | — | 29·0 | — | | |
| $F_2$ | — | 16·5 | — | 16·5 | 24·5 | 16·5 |
| $F_3$ | 25·0 | — | 25·0 | — | | |
| $F_4$ | 17·5, 26·5 | — | 22·0 | — | | |
| Uninj. MNC | | | | | | |
| $F_6$ | 19·0, 23·5, 26·5 | — | 23·0 | — | | |
| $F_{11}$ | 20·0 | — | 20·0 | — | 22·3 | 23·3 |
| $F_{12}$ | — | 23·0, 23·5 | — | 23·3 | | |
| Uninj. MCN | | | | | | |
| $F_5$ | 23·5 | 10·0 | 23·5 | 10·0 | 23·5 | 10·0 |
| Total " spontaneous " average age (hybrids) | | | | | 23·4 | 18·3 |
| MNC | | | | | | |
| $F_1$ | 20·5, 21·0 | 9·0 | 20·8 | 9·0 | | |
| $F_2$ | 20·0, 19·0, 24·5 | 14·5, 10·5 | 21·2 | 12·5 | | |
| $F_3$ | 19·5, 5·0 | — | 12·5 | — | | |
| $F_4$ | 20·0, 21·5, 20·0, 10·0, | 13·5, 4·0, 13·0 | 16·6 | 12·5 | | |
| | 19·0, 14·0, 12·0 | 19·5 | — | — | | |
| $F_5$ | 19·5, 14·0 | — | 16·8 | — | 17·1 | 12·2 |
| $F_7$ | 14·0, 16·0 | — | 15·0 | — | | |
| $F_8$ | 18·0 | — | 18·0 | — | | |
| $F_9$ | 15·0 | 13·5 | 15·0 | 13·5 | | |
| $F_{10}$ | 12·0, 21·0 | — | 16·5 | — | | |
| MCN | | | | | | |
| $F_1$ | 21·0, 23·0, 11·5, 14·5 | — | 17·5 | — | | |
| $F_2$ | 17·5, 16·0, 25·5 | 16·5 | 19·7 | 16·5 | | |
| $F_3$ | 26·5, 21·5, 26·5, 23·0 22·5, 18·5 | 17·0 | 23·1 | 17·0 | 19·8 | 12·1 |
| $F_4$ | 9·0 | 7·5, 7·5 | 9·0 | 7·5 | | |
| Total " induced " average age (hybrids) | | | | | 18·1 | 12·2 |

control solvents. They are tumours of infrequent occurrence which could readily escape observation unless the stomachs of all mice were submitted to routine examination at autopsy. Very occasional examples have been seen in recent years in this laboratory in the A and GFF strains. In the present case it happened that 2 inbred strains, NBT and CBA, were crossed, in which the incidences of this tumour, although small, differed significantly. In the reciprocal hybrids the incidence did not vary with the direction of the cross and was similar to that of the NBT strain—the parent strain with the higher incidence. The tumour occurs in middle and old age, and the observed incidence was therefore very closely connected with the average age of the mice at death, relative to the normal length of life of the strain. Thus the incidence in males was usually less than that in females, because the males tended to die sooner ; where they lived as long as the females the tumour-incidence was the same in both sexes. It is therefore believed that this tumour-incidence shows no real sex-difference, but because of the apparent sex-difference it has been thought more accurate to give the incidences in the two sexes separately and, in comparing two groups of mice, to compare females with females and males with males.

Although spontaneous malignant tumours of the forestomach were not seen in the two parent strains they have been known to occur occasionally in mice (Slye, Holmes and Wells, 1917 ; Wells, Slye and Holmes, 1938 ; Collins, Gardner and Strong, 1943 ; Jackson Memorial Laboratory, 1941). A few were found in the hybrids in the present experiment in mice which at no time had been, or could have been, exposed to methylcholanthrene (unless, as Beck (1952) suggested for benzpyrene, there had been some slight contamination of boxes with the carcinogen from previous occupants which had been injected) ; malignant tumours found in the $F_1$ mice must of course be suspect.

In spite of the rarity of the spontaneous gastric neoplasm, benign and malignant forestomach tumours have been produced experimentally in mice since 1927. Historical surveys of experimentally induced gastric tumours, up to 1940, have been given by Stewart (1940) and by Klein and Palmer (1941). Since then, Lorenz and Stewart (1940, 1948), Stewart (1941) and Stewart and Lorenz (1942, 1949) have published several reports on the induction of forestomach tumours by oral administration of carcinogens as well as by direct injection (the method by which they produced the first experimentally-induced glandular gastric carcinoma). Collins, Gardner and Strong (1943) induced benign and malignant forestomach tumours by intra-vaginal instillation and by oral administration of carcinogens, while Strong (1945) and Bagshaw and Strong (1950) obtained similar tumours in mice exposed to the carcinogen by remote injection, as in the present work. Although Gardner (1941) and Collins, Gardner and Strong (1943) believed that the tumours developed in those mice receiving the carcinogen by the vaginal route because the mice licked themselves and each other, thus swallowing some of the carcinogen which had oozed out, they obtained fewer tumours in this way than by direct oral administration ; and Bagshaw and Strong (1950) did not think that the small amount obtained by licking could account for the tumours produced in their experiment.

It has already been shown (Miller and Pybus, 1954*b*) that the control $F_1$ mice in the present experiment apparently obtained enough methylcholanthrene by licking their injected litter-mates to increase the lung tumour incidence to that in injected mice. Evidently the quantity of carcinogen thus received was not sufficient to raise the incidence of forestomach papillomata in the control $F_1$ mice above normal. The stomach tumour incidence in the injected mice was very much greater than that in the controls and the tumours appeared many months earlier, while, in addition to the increased proportion of tumorous mice, the number of papillomata per stomach was also greatly increased, and epidermoid carcinomata as well as sarcomata and mixed tumours were present in much greater numbers. There can be no doubt that the increase was due to the methylcholanthrene, but the effect in the present material did not persist in the uninjected descendants of the injected mice ; these uninjected mice had a tumour incidence similar to that of the controls, even when they were descended from 10 generations of injected animals.

The incidence of forestomach tumours in mice treated with carcinogens has been found to depend on the strain of mice. Strain differences were found by Gardner (1941), Collins, Gardner and Strong (1943) and by Lorenz and Stewart (1940, 1948), while Strong, Collins and Durand (1943) and Bagshaw and Strong (1950) found a difference between two sublines, one of which produced forestomach tumours and the other glandular gastric tumours. Susceptibility has been found to depend also on the carcinogen used as well as on its mode of administration. In the present work two inbred strains of mice with different incidences of spontaneous forestomach tumours were injected, but their susceptibilities to methylcholanthrene were the same ; admittedly the numbers of injected mice surviving to tumour age were small, the carcinogen used was one of the more potent (Klein and Palmer, 1941 ; Lorenz and Stewart, 1948), and the dose was large.

### SUMMARY.

1. Two inbred strains of mice, CBA and NBT, which differed in the incidence of spontaneous papillomata of the forestomach, were crossed reciprocally. A number of mice from the parent strains and from ten inbred generations of the hybrids each received one subcutaneous injection of 1·0 mg. methylcholanthrene at the age of two months.

2. The incidence of spontaneous forestomach papillomata in the hybrids did not vary with the direction of the cross and was the same as that of the more susceptible parent strain, the NBT, but the tumours appeared much later, as in the CBA strain. There was no real sex-difference in incidence, but only an apparent one caused by the earlier deaths of the males.

3. A few epidermoid carcinomata of the forestomach, presumed to be spontaneous, were seen in hybrids not exposed to the carcinogen, but none occurred in the parent strains.

4. In the injected groups the incidence of tumorous mice was greatly increased, the number of papillomata per stomach was also increased, and the tumours were produced many months earlier than in the controls. The incidence of malignant tumours of the forestomach (epidermoid carcinomata, sarcomata, and mixed carcino-sarcomata) was increased.

5. Injected members of the two inbred strains and the $F_1$ hybrids all had the same incidence of induced forestomach papillomata. In the later hybrid generations the induced tumour incidence fell as fewer mice survived to old age.

6. There was no evidence that the increased tumour incidence in the injected mice was maintained in their uninjected descendants, even after ten injected generations. The incidence in the uninjected descendants was similar to that in the controls.

This investigation was carried out with the aid of a research grant from the North of England Council of the British Empire Cancer Campaign. The authors would like to take this opportunity to make belated acknowledgment of a generous gift of methylcholanthrene received from Dr. G. M. Smith, of Yale University, during the early stages of this work at a time when supplies of the chemical were difficult to obtain in this country.

### Appendix

Statistics of significance for *significant* differences only.

| Index No. | Groups compared. | Difference. | Twice standard error. |
|---|---|---|---|
| 1 | NBT ♀ *v.* ♂ | 8·5 | 4·8 |
| 2 | NBT ♀ *v.* CBA ♀ | 9·3 | 4·7 |
| | NBT ♂ *v.* CBA ♂ | 3·3 | 2·4 |
| | NBT ♀+ ♂ *v.* CBA ♀ + ♂ | 6·3 | 5·1 |
| 3 | M/NBT ♀ *v.* ♂ | 77·3 | 17·9 |
| 4 | M/NBT ♀ *v.* NBT ♀ | 65·6 | 18·3 |
| 5 | M/NBT ♂ *v.* NBT ♂ | 3·3 | 2·4 |
| 6 | M/CBA ♀ *v.* ♂ | 85·7 | 26·5 |
| 7 | M/CBA ♀ *v.* CBA ♀ | 97·7 | 2·0 |
| 8 | M/NBT $F_1$ ♀ *v.* ♂ | 14·26 | 14·16 |
| 9 | M/NBT $F_1$ ♀ *v.* NBT ♀ | 17·7 | 11·8 |
| | M/NBT $F_1$ ♂ *v.* NBT ♂ | 11·9 | 9·2 |
| 10 | M/NBT $F_1$ ♀ *v.* M/NBT ♀ | 47·9 | 21·9 |
| 11 | M/CBA $F_1$ ♀ *v.* M/CBA ♀ | 95·7 | 3·2 |
| 12 | M/NBT *v.* NBT | 29·8 | 13·1 |
| 13 | MNC $F_2$ ♀ *v.* ♂ | 14·4 | 12·2 |
| | MCN $F_1$ ♀ *v.* ♂ | 27·8 | 24·8 |
| | MCN $F_2$ ♀ *v.* ♂ | 27·3 | 15·5 |
| 14 | MNC $F_1$ ♀ *v.* MNC $F_2$ ♀ | 27·9 | 19·6 |
| | (♂ significant by inspection) | | |
| | MCN $F_1$ ♀ *v.* MCN $F_2$ ♀ | 30·5 | 19·8 |
| | MCN $F_1$ ♂ *v.* MCN $F_2$ ♂ | 30·0 | 21·5 |
| 15 | MNC $F_2$ ♀ + ♂ *v.* MCN $F_2$ ♀ + ♂ | 11·5 | 10·3 |
| | MNC $F_2$ ♀ *v.* MCN $F_2$ ♀ | 16·4 | 15·0 |
| 16 | MNC $F_1$ *v.* NC $F_1$ | 43·8 | 17·2 |
| | MNC $F_2$ *v.* NC $F_2$ | 10·4 | 7·8 |
| | MCN $F_1$ *v.* CN $F_1$ | 39·3 | 14·7 |
| | MCN $F_2$ *v.* CN $F_2$ | 26·9 | 9·1 |
| 17 | MNC $F_1$ *v.* NC $F_1$ | 33·1 | 31·2 |
| | MNC $F_2$ *v.* NC $F_2$ | 30·4 | 17·6 |
| | MCN $F_1$ *v.* CN $F_1$ | 51·4 | 25·7 |
| | MCN $F_2$ *v.* CN $F_2$ | 43·5 | 21·3 |
| 18 | NC ♀ *v.* ♂ (total) | 5·1 | 3·3 |
| 19 | CN $F_9$ ♀ *v.* ♂ | 10·0 | 9·5 |
| | CN $F_{11}$ ♀ *v.* ♂ | 7·1 | 6·9 |
| | CN ♀ *v.* ♂ (total) | 3·0 | 2·99 |
| 20 | MNC $F_4$ ♀ *v.* ♂ | 19·4 | 12·9 |
| | MNC $F_5$ ♀ *v.* ♂ | 28·3 | 13·7 |
| | MNC $F_7$ ♀ *v.* ♂ | 13·6 | 8·9 |
| | MNC $F_8$ ♀ *v.* ♂ | 21·8 | 16·7 |
| | MNC $F_{10}$ ♀ *v.* ♂ | 10·9 | 10·1 |
| | MNC ♀ *v.* ♂ (total) | 15·2 | 4·4 |
| 21 | MCN $F_3$ ♀ *v.* ♂ | 38·1 | 17·9 |
| | MCN ♀ *v.* ♂ (total) | 13·1 | 5·3 |
| 22 | MNC ♀ *v.* NC ♀ | 17·1 | 4·5 |
| | MNC ♂ *v.* NC ♂ | 7·0 | 3·2 |
| | MCN ♀ *v.* CN ♀ | 18·7 | 4·7 |
| | MCN ♂ *v.* CN ♂ | 8·6 | 3·8 |
| 23 | uninj. MNC $F_2$ ♀ *v.* ♂ | 5·8 | 3·98 |
| | uninj. MNC ♀ *v.* ♂ (total) | 4·9 | 2·4 |
| 24 | uninj. MCN ♀ *v.* uninj. MNC ♀ | 7·4 | 2·4 |
| | uninj. MCN ♂ *v.* uninj. MNC ♂ | 2·9 | 1·8 |
| | uninj. MCN ♀ *v.* CN ♀ | 6·6 | 2·6 |
| | uninj. MCN ♂ *v.* CN ♂ | 4·0 | 2·3 |

### REFERENCES.

BAGSHAW, M. A. AND STRONG, L. C.—(1950) *J. nat. Cancer Inst.*, **11**, 141.
BECK, S.—(1952) *Ann. Rep. Brit. Emp. Cancer Campgn.*, **30**, 253.
BONNÉ, C.—(1927) *Z. Krebsforsch.*, **25**, 1.
COLLINS, V. J., GARDNER, W. U. AND STRONG, L. C.—(1943) *Cancer Res.*, **3**, 29.
FIRMINGER, H. I. AND STEWART, H. L.—(1951) *J. nat. Cancer Inst.*, **12**, 491.
GARDNER, W. U.—(1941) *Ibid.*, **1**, 502.
KLEIN, A. J. AND PALMER, W. L.—(1941) *Ibid.*, **1**, 559.
LORENZ, E. AND STEWART, H. L.—(1940) *Ibid.*, **1**, 273.—(1948) *Ibid.*, **9**, 173.
MILLER E. W. AND PYBUS, F. C.—(1954*a*) *Brit. J. Cancer*, **8**, 163.—(1954*b*) *Ibid.*, **8**, 466.—(1954*c*) *Ibid.*, **8**, 655.
PEACOCK, P. R., BECK, S. AND CHALMERS, J. G.—(1953) *J. nat. Cancer Inst.*, **13**, 931.
SLYE, M., HOLMES, H. F. AND WELLS, H. G.—(1917) *J. Cancer Res.*, **2**, 401.
Jackson Memorial Laboratory, Staff of—(1941) 'Biology of the Laboratory Mouse.' Philadelphia (The Blakiston Co.), p. 219.
STEWART, H. L.—(1940) *Arch. Path.*, **29**, 153.—(1941) *J. nat. Cancer Inst.*, **1**, 489.
*Idem* AND ANDERVONT, H. B.—(1938) *Arch. Path.*, **26**, 1009.
*Idem* AND LORENZ, F.—(1942) *J. nat. Cancer Inst.*, **3**, 175.—(1949) *Ibid.*, **10**, 147.
STRONG, L. C.—(1945) *Ibid.*, **5**, 339.
*Idem*, COLLINS, V. J. AND DURAND, E. A.—(1943) *Cancer Res.*, **3**, 21.
WELLS, H. G., SLYE, M. AND HOLMES, H. F.—(1938) *Amer. J. Cancer*, **33**, 223.